# 場面緘黙児・者の発話評価：システマティック・レビュー

**DOI:** 10.12688/f1000research.113302.1

**Published:** 2022-07-28

**Authors:** 藤間友里亜 藤間, 松田壮一郎 松田

**Affiliations:** 1 Behavioral Design Laboratory, University of Tsukuba, Tsukuba, Japan; 2 Faculty of Human Sciences, University of Tsukuba, Tsukuba, Japan

**Keywords:** selective mutism, anxiety disorder, neurodevelopmental disorder, systematic review, assessment, speech

## Abstract

**背景：**場面緘黙の主要な特徴は特定の社会的状況における発話の欠如である。したがって，場面緘黙の診断確定，場面緘黙と他の障害との鑑別のため，異なる社会的状況での発話評価が重要である。しかしながら，場面緘黙児・者の発話評価手法は未だ確立されておらず，直接行動を観察する評価手法は少ない。更に，異なる社会的状況における場面緘黙児・者の発話の評価方法について系統的なレビューを行った研究はこれまでにない。本システマティック・レビューの目的は，先行研究において，場面緘黙の主症状である特定の社会的状況における発話の欠如がどのように評価されてきたか，整理することだった。

**方法：**Web of Science，PsycINFO，PubMedの3つのデータベースを使用し，2020年1月28日に系統的検索を行った。場面緘黙児・者を対象とした実証データを報告した調査・実験研究をレビューの対象とした。展望論文，質的研究，疫学研究，介入研究は除外した。診断基準，場面緘黙診断確定手法，場面緘黙と他の障害との鑑別手法，発話評価手法について整理した。

**結果：**合計447編の研究についてスクリーニングを行い，採用基準に合致した研究は60編だった。場面緘黙診断を確定するため，様々な面接や質問紙が使用されていた。しかし，多くの面接や質問紙は妥当性が検証されていなかった。場面緘黙診断確定に関して妥当性検証済みの発話評価手法を用いた研究は2/60編のみだった。また，場面緘黙と他の障害を鑑別する評価手法は研究間で一致していなかった。17編の研究は，診断確定以外の目的で発話を測定していた。そのほとんどの研究（16/17編）で場面緘黙の重症度を評価するため，質問紙が使用されており，行動観察を行った研究は1編のみだった。実生活での発話測定に基づく場面緘黙児・者の評価手法は確立されていなかった。

**結論：**本研究は，介入研究をレビューしていないという限界がある。しかし，本研究によって，場面緘黙の調査・実験研究において発話評価手法が確立されていないという問題が明らかになった。今後の研究では，場面緘黙診断確定ため，また，場面緘黙と他の障害との鑑別のため，異なる社会的状況における発話評価手法を確立する必要がある。

## Abstract (en)

**Background:** The main characteristic of selective mutism (SM) is the failure to speak in specific social situations. Thus, assessing speech across social contexts is important for confirming a diagnosis of SM and for differentiating it from other disorders. The purpose of this review was to organize how the core symptom of SM, a lack of speech in specific social situations, has been assessed in previous studies.

**Methods:** A systematic search of articles was conducted in three databases, Web of Science, PsycINFO, and PubMed and reviews of surveys or experimental studies that reported empirical data on individuals with SM were performed. We excluded review, qualitative, epidemiological, and intervention studies. The study summarized the diagnostic criteria, methods of confirming SM diagnosis, distinction of SM from other disorders, and methods of speech assessment.

**Results:** A total of 447 articles were screened, where 60 articles were considered eligible. The results demonstrate that different interviews and questionnaires were used to establish the diagnosis of SM. However, the majority of interviews and questionnaires lacked validation. Only two (2/60) articles used validated methods of speech assessment to confirm SM diagnosis. Moreover, a consensus was lacking on the assessment method for differentiating SM from other disorders across studies. Specifically, 17 studies measured speech and are not intended for diagnosis. The majority of studies (16/17) used the questionnaire to assess the severity of the SM condition, and only one study conducted behavioral observation. Assessment methods based on the measurement of speech in real-life situations for individuals with SM were not established.

**Conclusion:** We have the limitation that we did not review intervention studies. However, this systematic review revealed the problem that speech assessment methods for surveys or experimental studies of SM were not established. Future studies should establish methods of speech assessment across social situations to assess SM symptoms.

## I. 序論

場面緘黙 (Selective Mutism) は，「他の状況で話しているにもかかわらず，話すことが期待されている特定の社会的状況（例 : 学校）において話すことが一貫してできない」 ( American Psychiatric Association, 2013 髙橋・大野監訳，2014, p.193) ことによって特徴づけられる，不安症の一つであり，その有病率は 0.03-0.79% である ( Driessen, Blom, Muris, Blashfield, & Molendijk, 2020)。ある特定の社会的状況において話す能力がある場合に限り，場面緘黙の診断が下されるため，特定の社会的状況に発話の障害が限定されないコミュニケーションの障害（ *e.g.*, 言語症，語音症，小児期発症流暢症，社会的コミュニケーション症などのコミュニケーション症，自閉スペクトラム症，知的能力障害などの神経発達症，統合失調症やその他の精神病性障害）とは区別される。Diagnostic and Statistical Manual of Mental Disorders, Fifth Edition　(DSM-5) において，場面緘黙は不安症群に位置づけられているが，不安症群内の他の障害（特に社交不安症）との鑑別や，他の障害群である神経発達症群の自閉スペクトラム症等との鑑別方法が確立されていないことが指摘されている ( Driessen *et al.*, 2020; Steffenburg, Steffenburg, Gillberg, & Billstedt, 2018)。

場面緘黙とその他の障害の併存についても，これまで数多く指摘されてきた。場面緘黙と社交不安症，自閉スペクトラム症の症状の併存について指摘した先行研究は複数存在しており ( Driessen *et al.*, 2020; Steffenburg *et al.*, 2018)，場面緘黙児・者における社交不安症の併存率を調べた22編の研究を対象としたメタ分析では場面緘黙児・者の社交不安症の併存率が，0%-100%までのばらつきを示しており，平均 69% だったことを報告している ( Driessen *et al.*, 2020)。社交不安症の特徴である社交場面に対する恐怖は場面緘黙児・者も示すことがあるものの，場面緘黙は，特定状況下での発話の一貫した欠如が診断の基準に含まれている点が社交不安症と異なる。さらに，場面緘黙児・者の 62.9% が自閉スペクトラム症の特徴を有するという報告もある ( Steffenburg *et al.*, 2018)。自閉スペクトラム症児・者も社会的コミュニケーションの問題を示すが，場面緘黙児・者では社会的コミュニケーションの障害が特定状況下に限られる点で異なる。以上から，場面緘黙と他の障害との鑑別や，他の障害の併存を同定するためには，異なる社会的状況下での発話評価が重要である。

数十年に及ぶ場面緘黙児・者を対象とした先行研究のほとんどは，様々な質問紙や面接を用いて，社交不安症や自閉スペクトラム症など他の障害との鑑別のため，発話を評価してきた。しかし，質問紙や面接による評価では，複数の社会的状況における発話の生起頻度やその形態に関するデータを直接収集していないため，信頼性に問題があることが考えられる。質問紙や面接の回答は事実そのものではなく回答者の認識であり，回答者の主観的な推論の影響を受けるという問題が指摘されている ( Richardson, 2004)。場面緘黙児・者を対象とした研究においては，頑健な手法による，異なる社会的状況における発話の評価が重要である。それにもかかわらず，異なる社会的状況における発話の評価手法についての系統的な整理は，ほとんど行われていない。

これまで場面緘黙児・者を対象とした研究のシステマティック・レビューでは，介入方法や介入効果に関するものがほとんどだった ( Cohan, Chavira, & Stein, 2006; Manassis, Oerbeck, & Overgaard, 2016; 水野・関口・臼倉，2018; Østergaard, 2018; Zakszeski & DuPaul, 2017)。場面緘黙の主症状の評価手法に関する系統的な整理を行った研究は近年出版された ( Rodrigues Pereira, Ensink, Lindauer, De Jonge, & Utens, 2021)。 Rodrigues Pereira *et al. *(2021) では場面緘黙のスクリーニング及び診断に用いられたツールに焦点を当て，各ツールの長所・短所について考察を行っている。しかし，先行研究で用いられた診断基準の整理や，他の診断との鑑別がどのように行われたかについての検討はされていなかった。本研究の目的は，場面緘黙児・者を対象とした調査・実験研究において，場面緘黙診断の確定方法や，場面緘黙と他の障害との鑑別手法も含め，発話がどのように評価されてきたかを整理することだった。また，レビュー結果に基づき，異なる社会的状況での発話について，信頼性の高い客観的な評価を行うための課題について考察することも目的とした。

## II. 方法

### 1. リサーチクエスチョン

優れたリサーチクエスチョンが満たすべき FINER (Feasibility; 実施可能性，Interesting; 科学的興味深さ, Novel; 新規性, Ethical; 倫理性, Relevant; 必要性)の基準 ( Hulley, Cummings, Browner, Grady, & Newman, 2013 木原・木原訳，2014, p.19–21) を考慮し，実証的な研究において，場面緘黙児・者の発話はどのように評価されてきたか，をリサーチクエスチョンとした。

### 2. 論文の選定基準

英語で記述された場面緘黙児・者を対象とした実証データに基づく調査・実験研究をレビューの対象とした。英語以外の言語で記述された文献，展望論文，質的研究，疫学研究，介入研究は除外した。

### 3. 論文の選定

レビューの対象とする論文は，システマティック・レビューの方法の国際的規範となっている PRISMA の手順 ( Liberati *et al.*, 2009) に従い，選定した。Web of Science，PsycINFO，PubMed の 3 つのデータベースを使用し，論文の検索を行った。論文タイトルを検索対象とし， “selective mutism” OR “elective mutism” を検索キーワードとした。加えて，Web of Science ではドキュメントタイプを article に絞り込み，PsycINFO では Peer Reviewed Journal を条件として絞り込みをした。検索日は 2020 年 1 月 28 日であり，検索日までに出版された論文を対象とした。さらに過去に場面緘黙児・者を対象としたシステマティック・レビュー論文 ( Kristensen, 1997; Muris & Ollendick, 2015; Sharp, Sheman, & Gross, 2007) においてレビューされている論文を加えた。レビューの対象とする論文を決定するため，2 名の著者が独立にスクリーニングを行った。1 段階目のスクリーニングとして題目と抄録に基づくスクリーニングを行った。著者間で判断に相違があった論文は2段階目のスクリーニングに含めることとした。2 段階目のスクリーニングとして本文全体に基づくスクリーニングを行った。著者間で判断に相違があった場合には，著者間で協議を行い，最終的にレビュー対象へ含めるかどうか決定した。

### 4. 情報の抽出と統合

情報の抽出と統合は，第一著者が行った上で，第二著者と協議の上，最終的に論文へ含める情報を決定した。本研究では，発話評価手法について概観することを目的としため，発話評価に関する内容として，(1) 診断基準，(2) 場面緘黙診断確定手法，(3) 場面緘黙と他の障害との鑑別方法，(4) その他の発話評価手法，に関する情報を抽出した。(1) 診断基準に関しては，用いられた基準が明記されていた研究数，各診断基準を用いた研究数について調べた。(2) 場面緘黙診断確定方法に関しては，診断を確定するために用いた方法が明記されていた研究数を示し，場面緘黙診断確定を目的として開発された手法について，測定法の種類ごとに用いられた研究数及び用いられた尺度について調べた。(3) 場面緘黙と他の障害との鑑別方法に関しては，場面緘黙群と他の障害群の群間比較研究を対象に，鑑別方法が明記されていた研究数，用いられた測定法の種類及び尺度，場面緘黙と他の障害の併存が認められた場合の群の割り当てについて調べた。(4) その他の発話評価手法に関しては，診断確定以外の目的で発話評価を行った研究を対象に，用いられた測定法の種類ごとに用いられた研究数，用いられた尺度について調べた。

## III. 結果

### 1. レビュー対象論文の選定結果

**Figure 1.**
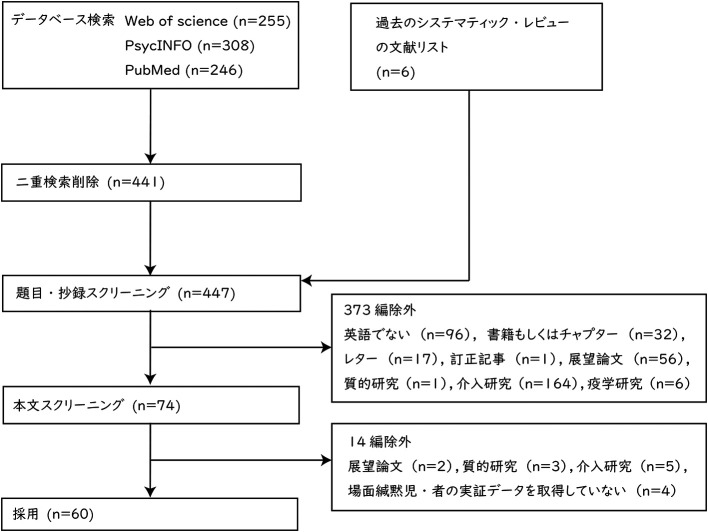
PRISMA に基づいた論文選定フロー.

レビューの対象とする論文の選定結果を Figure 1 に示した。データベースによる検索の結果は，Web of Science が 255 編，PsycINFO が 308 編，PubMed が 246 編であった。二重検索を削除した結果，441 編になった。これ以外に過去のシステマティック・レビューの論文 ( Kristensen, 1997; Muris & Ollendick, 2015; Sharp *et al.*, 2007) においてレビューされている論文の中から 6 編を加え，合計 447 編となった。447 編の論文について，題目と抄録の内容によってスクリーニングを行った。英語以外の言語で記述された文献 96 編，書籍もしくはチャプター 32 編，レター 17 編，訂正記事1編，展望論文 56 編，質的研究 1 編，介入研究 164 編，疫学研究6編，合計 373 編を除外した。次に，残りの 74 編について本文全体の内容に基づき，スクリーニングを実施した。展望論文2編，質的研究3編，介入研究5編，場面緘黙児・者の実証データを取得していない研究 4 編，合計 14 編を除外した。最終的なレビュー対象の論文は 60 編だった。

### 2. 用いられた医学的診断基準

医学的診断基準を Table 1 に示した。レビュー対象のうち，使用した医学的診断基準が明記されていた論文は 44/60 編だった。単独で使用された診断基準の内訳は，DSM-5 (3 編)，DSM-IV-TR (7 編)，DSM-IV (25 編),　DSM-III-R (2 編)，ICD-10 (3 編) だった。ただし， Cleator and Hand (2001) では，DSM-IV を用いていたものの，診断基準の A–D を満たすことを条件とし，診断基準Eは考慮されなかった。複数の診断基準を用いた研究では，DSM-IVとICD-10 (1 編)，DSM-III-R と DSM-IV (2 編)，ICD-9 と ICD-10 (1 編) の併用が認められた。

**Table 1.** 場面緘黙(選択性緘黙)の診断基準.

DSM-III	A.学校を含むほとんど全ての社会的場面において，喋ることを持続的に拒否すること。 B.話された言葉を理解し，話す能力の存在。 C.他の精神障害もしくは身体疾患に起因しない。
DSM-III-R	A.1 つ，またはより多くの社会的場面（学校を含む）において，話すことの持続的拒否。 B.話し言葉を理解し，話す能力の存在。
DSM-IV	A.他の状況では話すことができるにもかかわらず，特定の社会状況（話すことが期待されている状況，例えば，学校）では，一貫して話すことができない。 B.この障害が学業上，職業上の成績，または社会的な意思伝達を妨害している。 C.この障害の持続期間は少なくとも 1 カ月（学校での最初の 1 カ月に限定されない）。 D.話すことができないことは，その社会状況で要求されるまたは快適な，話し言葉を知らないことによるものではない。 E.この障害はコミュニケーション障害（例:吃音症）では上手く説明されないし，また，広汎性発達障害，精神分裂病，またはその他の精神病性障害の経過中にのみ起こるものではない。
DSM-IV-TR	A.他の状況では話すことができるにもかかわらず，特定の社会的状況(話すことが期待されている状況，例:学校)では，一貫して話すことができない。 B.この障害が，学業上，職業上の成績，または対人的コミュニケーションを妨害している。 C.この障害の持続期間は少なくとも1カ月（学校での最初の1カ月に限定されない） D.話すことができないことは，その社会状況で要求される話し言葉の楽しさや知識がないことによるものではない。 E.この障害はコミュニケーション障害（例:吃音症）ではうまく説明されないし，また，広汎性発達障害，統合失調症，または他の精神病性障害の経過中にのみ起こるものではない。
DSM-5	A.他の状況で話しているにもかかわらず，話すことが期待されている特定の社会的状況（例:学校）において，話すことが一貫してできない。 B.その障害が，学業上，職業上の成績，または対人的コミュニケーションを妨げている。 C.その障害の持続期間は，少なくとも 1 カ月（学校の最初の 1 カ月だけに限定されない）である。 D.話すことができないことは，その社会的状況で要求されている話し言葉の知識，または話すことに関する楽しさが不足していることによるものではない。 E.その障害は，コミュニケーション症（例:小児期発症流暢症）ではうまく説明されず，また自閉スペクトラム症，統合失調症，または他の精神病性障害の経過中にのみ起こるものではない。
ICD-10	A.言語表現および言語理解は，標準化された検査を個別に施行して，その小児の年齢での 2 標準偏差以内にあること。 B.別の状況では話しているにもかかわらず，話すことを求められるような社会的状況下（たとえば，学校）では一貫して話せないという，明白な証拠があること。 C.4 週間以上持続すること。 D.広汎性発達障害 (F84.-) がないこと。 E.そのような社会的状況で必要な話しことばについての知識がないために，話せないというわけではないこと。

### 3. 場面緘黙の診断確定に用いられた手法

診断基準，診断を確定するために用いられたと論文中で記載された測定法，測定法の種類について，出版年の新しい順に示した ( Table 2)。場面緘黙の診断確定に用いられた測定法について，記載していた研究は 44/60 編あり，複数の方法の併用や医療記録によって総合的に判断した研究が最も多かった (24 編)。場面緘黙の診断を確定する目的で開発された測定法を使用した研究と，他の目的で開発された測定法を場面緘黙診断確定のために使用した研究があった。場面緘黙の診断を確定する目的で開発された測定法は Table 2 中に太字で示した。測定法の種類については，場面緘黙診断確定を目的として開発された測定法の種類を示した。診断を確定する測定法として面接 (22 編)，質問紙 (7 編)が用いられていた。

場面緘黙の診断確定のために面接を用いた研究のほとんど (14/22 編) で，Anxiety Disorders Interview Schedule for DSM-IV (ADIS-IV; Silverman & Albano, 1996) が用いられていた。 ADIS-IVは，DSM-IVの診断基準に基づき，不安症と不安関連症の診断確定を行うための半構造化面接である。保護者をインフォーマントとする ADIS-IV-Parent version (ADIS-IV-P) と子ども本人をインフォーマントとする ADIS-IV-Children version (ADIS-IV-C) がある。ADIS-IV-P/C は Lyneham, Abbott, and Rapee (2007)， Silverman, Saavedra, and Pina (2001)， Wood, Piacentini, Bergman, McCracken, and Barrios (2002) で，一部の障害診断確定に関して妥当性検証は行われていたが，これらの研究では対象者の中に場面緘黙と診断された人々が含まれておらず，場面緘黙診断確定の妥当性は確認されていなかった。ADIS-IV-P は，Yes，No，Other で回答される診断確定のための質問（ *e.g.*, 彼または彼女は友人やその他の人々の質問に答えることを拒否しますか，彼または彼女は家庭で家族と一緒にいるときに話しますか）が 8 つ，9 段階で回答される重症度評価のための項目（この問題はあなたの子どもの生活をどの程度妨げていますか）が1つある。質問には，DSM-IVの診断基準 A, B, C に対応する項目が含まれている。診断基準 D （話していないことは，要求される話し言葉，快適な話し言葉の知識の不足によるものではない）に対応する項目は含まれていない。ADIS-IV-C では，場面緘黙の項目は，Screening Questions for Additional Childhood Disorders の中に含まれており，この面接の結果，場面緘黙の可能性が考えられる場合には更に詳細を確認する必要がある。レビュー対象に含まれた ADIS-IV-C を行った研究では，保護者版の ADIS-IV-P も併せて実施されていた。

ADIS-IVの他に用いられた診断確定のための面接には，Kinder-Version des Diagnostischen Interviews für Psychische Störungen (Kinder-DIPS; Adornetto, In-Albon, & Schneider, 2008) (3/22 編)，Diagnostic Interview for Children and Adolescents-IV (DICA-IV; Reich, Welner, Herjanic, & Multihealth Systems staff, 1997) (2/22 編)，Parent as Respondent Informant Schedule (PARIS; Klein & Mannuzza, 1992) (2/22 編)，Brief Child and Family Phone Interview (BCFPI; Cunningham, Boyle, Hong, Pettingill, Bohaychuck, 2009) (1/22 編)があった。Kinder-DIPS と DICA-IV は子ども本人と保護者，BCFPI 及び PARIS は保護者をインフォーマントとする。Kinder-DIPS は DSM-IV-TR 及び ICD-10 に，DICA-IV は DSM-IV 及び DSM-III-R，PARIS は DSM-III-R にそれぞれ対応している。Kinder-DIPS，DICA-IV, PARIS はそれぞれ小児精神疾患の診断確定を目的として開発された一方，BCFPI は，感情・行動上の問題を査定する目的で開発された。Kinder-DIPS は Adornetto *et al.* (2008) で，DICA-IV は Reich (2000) で，BCFPI は Cunningham *et al.* (2009) で，一部の障害診断確定に関して妥当性検証が行われていたものの，これらの研究では対象者の中に場面緘黙と診断された人々が含まれておらず，場面緘黙診断確定の妥当性は確認されていなかった。PARIS について妥当性を検証した研究は著者らの知る限り，執筆時点で存在しなかった。

場面緘黙の診断確定に用いられた質問紙には，Speech Situations Questionnaire ( Cunningham, McHolm, Boyle, & Patel 2004) (5/7 編)とFrankfurt Scale of Selective Mutism (FSSM; Gensthaler, *et al.*, 2020) (2/7 編)の診断尺度があった。Speech Situations Questionnaire は保護者が回答する Speech Situations Questionnaire-P と教師が回答する Speech Situations Questionnaire-T がある ( Nowakowski *et al.*, 2011)。Speech Situations Questionnaire-P は，家庭，学校，地域社会での子どもの発話頻度を 3 件法で尋ねる 15 項目の質問紙である。Speech Situations Questionnaire-T は，教室，廊下，運動場など学校内の様々な場所での子どもの発話頻度を 3 件法で尋ねる 7 項目の質問紙である。Speech Situations Questionnaire-P/T について妥当性を検証した研究は著者らの知る限り，執筆時点で存在しない。

FSSM は， 場面緘黙にあてはまるかを評価する診断尺度と場面緘黙の重症度を評価する重症度尺度から構成されている，保護者回答の質問紙である。診断尺度では，10 項目（ *e.g.*, 話すことが期待される特定の状況で話していない，家での話し方と外での話し方に明らかな違いがある）について「はい」か「いいえ」で回答を求める。FSSM 診断尺度は Gensthaler *et al.* (2020) で妥当性検証が行われている。

### 4. 場面緘黙と他の障害との鑑別に用いられた手法

定型発達群以外の対照群を設定し，群間比較を行った研究 (27 編) では，様々な障害群を対照群としていた。単一の障害で構成される対照群で最も多かったのは社交不安症群 (10/27 編) だった。場面緘黙以外の複数の不安症を対照群として設定した研究(10/27編)も多かった。また，不安障害に限らず，複数の疾患を一つの対照群として設定する研究 (6/27 編) もあった。その他には，全般不安症 (1/27 編)，場面緘黙と自閉スペクトラム症の併存 (1/27 編)，不安症と ADHD の併存 (1/27 編)も対照群として用いられていた。対照群の障害・疾患が不明な研究 (2/27 編) もあった。

対照群を設定した研究では，診断の鑑別方法を記載していた研究が過半数 (18/27 編) だったが，記載していない研究もあった (9/27 編)。社交不安症群を対照群に含む研究 (10 編) では，診断を確定するために半構造化面接 (8/10 編)もしくは質問紙 (2/10 編) が用いられていた。半構造化面接を実施し場面緘黙群と社交不安症群を群分けした 8 編の研究では， Kinder-DIPS，ADIS-IV-C/P， DICA-IVが実施されていた。場面緘黙と社交不安症の併存が認められた場合には，場面緘黙の基準が優先され，場面緘黙群に含まれる手続きを用いた研究が多かった (5/8 編)。残り3編の研究では，両障害が併存した場合の手続きについて記述されていなかった。診断の確定に質問紙を使用した 2 編の研究では，場面緘黙の診断確定のために FSSM，社交不安症の診断確定のために Social Phobia and Anxiety Inventory for Children German Version (SPAI-C; Melfsen, Walitza, & Warnke, 2011) が使用されていた。質問紙を使用した2編の研究では，場面緘黙と社交不安症が併存していた場合，場面緘黙の診断が優先されていた。

場面緘黙以外の不安症群を対照群に含む研究 (10 編) には，半構造化面接のみによって群分けを行った研究が 5/10 編，半構造化面接と質問紙の併用によって群分けを行った研究が 4/10 編，群分けの方法について記載のない研究が 1/10 編あった。半構造化面接のみを行った研究では ADIS-IVが実施されていた (1 編は Parent Version のみ，4 編は Child Version と Parent Version)。半構造化面接と質問紙を用いた研究には C-DISC-IV と Speech Situations Questionnaire を実施した研究が3編，C-DISC-IVと Speech Situations Questionnaire に加えて BCFPI を実施した研究が1編あった。不安症群を対照群とする研究においても，場面緘黙と他の不安症が併存する場合，場面緘黙の診断が優先し，場面緘黙群に含める研究が多かった (7 編)。障害が併存していた場合の手続きについて記載がない研究もあった (2 編)。

場面緘黙と自閉スペクトラム症の併存群と場面緘黙群を比較した唯一の研究では，臨床面接，保護者対象の面接，質問紙 (DSM-IVチェックリスト，保護者または教師回答の質問紙，Autism Spectrum Screening Questionnaire) に基づいて医師が臨床心理士と相談した上で判断していた。対象児の示す症状が場面緘黙か自閉スペクトラム症のどちらかだけで説明できない場合に，2 つの障害を併存していると判断された。

**Table 2.** 場面緘黙診断確定に用いられた手法.

研究	診断基準	診断確定のために用いられた方法（場面緘黙の特定を目的として開発された方法は太字）	本人面接	保護者面接	保護者質問紙	教師質問紙
Gensthaler *et al.* (2020)	DSM-IV-TR	**Kinder-DIPS**		✓		
Vogel *et al.* (2019)	-	**FSSM**			✓	
Schwenck *et al.* (2019)	-	**FSSM**			✓	
Klein *et al.* (2019)	DSM-5	**ADIS-IV-C/P** BASC-3 SMQ 構造化された発達面接	✓	✓		
Steffenburg *et al.* (2018)	DSM-IV	保護者面接（言語発達，場面緘黙症状の開始，診断された年齢，家庭でスウェーデン語以外の言語にさらされているか） 臨床アセスメント（DSM-IV チェックリスト，FTF，ASSQ)				
Capozzi *et al.* (2018)	DSM-IV-TR，DSM-5	K-SADS-PL (DSM-III-R，DSM-IV)				
Gensthaler, Maichrowitz *et al.* (2016)	DSM-IV-TR	**Kinder-DIPS**		✓		
Gensthaler, Khalaf *et al.* (2016)	DSM-IV-TR	**Kinder-DIPS**		✓		
Diliberto & Kearney (2016)	DSM-IV	**ADIS-IV-P**		✓		
Martinez *et al.* (2015)	DSM-IV	**ADIS-IV-P**		✓		
Muchnik *et al.* (2013)	DSM-IV-TR	K-SADS-PL (DSM-III-R，DSM-IV) 家庭場面の録画・録音				
Levin‐Decanini *et al.* (2013)	DSM-IV	一般的な健康評価面接 **ADIS-IV-C/P**	✓	✓		
Klein *et al.* (2013)	DSM-IV-TR	視聴覚検査 BASC-2 セラピストが作成したDSM-IVの診断基準に基づく構造化された質問紙				
Alyanak *et al.* (2013)	DSM-IV	包括的アセスメント （Dow et al. のガイドラインに従った） 非標準化臨床面接				
Young *et al.* (2012)	-	**ADIS-IV-P** C-GAS		✓		
Heilman *et al.* (2012)	-	**ADIS-IV-P**		✓		
Nowakowski *et al.* (2011)	-	**Speech Situations Questionnaire-P/T**			✓	✓
Edison *et al.* (2011)	DSM-IV，ICD-10	**BCFPI** **Speech Situations Questionnaire-P/T** （どちらか一方）		✓	✓	✓
Henkin *et al.* (2010)	DSM-IV-TR	半構造化臨床面接 SMQ SPAI-C SCARED 家庭場面の録画・録音				
Carbone *et al.* (2010)	-	**Speech Situations Questionnaire-P/T**			✓	✓
Nowakowski *et al.* (2009)	-	**Speech Situations Questionnaire-P/T**			✓	✓
Letamendi *et al.* (2008)	DSM-IV	**ADIS-IV-C/P**	✓	✓		
Cohan *et al.* (2008)	-	**ADIS-IV-P**		✓		
Bergman *et al.* (2008) 研究 2	-	包括的評価 **ADIS-IV-C/P** （参加者の 92% 対象）	✓	✓		
Manassis *et al.* (2007)	DSM-IV	**ADIS-IV-C/P**	✓	✓		
Chavira *et al.* (2007)	DSM-IV	**ADIS-IV-C/P** SMQ	✓	✓		
Arie *et al.* (2007)	DSM-IV-TR	半構造化臨床面接 実験室での保護者と相互作用中の発話行動の観察 実験室での実験者と相互作用中の発話行動の観察 家庭場面の録画・録音				
Yeganeh *et al.* (2006)	DSM-IV	ADIS-IV-C/P	✓	✓		
Kristensen & Oerbeck (2006)	DSM-IV	紹介元のセラピストとの話し合い 構造化面接 直接観察				
Cunningham *et al.* (2006)	-	面接 Speech Situations Questionnaire-P/T			✓	✓
Vecchio & Kearney (2005)	DSM-IV	ADIS-IV-C/P	✓	✓		
McInnes *et al.* (2004)	DSM-IV	半構造化面接 DICA-IV (structured conputerized version)		✓		
Cunningham *et al.* (2004)	DSM-IV	面接 保護者評価				
Bar-Haim *et al.* (2004)	DSM-IV	半構造化面接 家庭場面の録画				
Yeganeh *et al.* (2003)	DSM-IV	ADIS-IV-C/P	✓	✓		
Manassis *et al.* (2003)	DSM-IV	DICA-IV (semistructured interview) DICA-IV (structured conputerized version)		✓		

### 5. その他の発話評価手法

診断確定以外の目的で発話評価を行った研究は 17/60 編あった。診断確定以外の目的で用いられた発話評価の手法を出版年の新しい順に示した ( Table 3)。発話評価の方法として質問紙 (16 編)，面接 (1 編)，観察 (1 編) が用いられていた。質問紙の回答者は，保護者 (14 編)，教師 (2 編)，場面緘黙児・者本人 (2 編) だった。保護者回答の質問紙を用いた研究 3/14 編，場面緘黙児・者回答の質問紙を用いた研究 2/2 編では，それぞれの研究オリジナルの質問紙を用いていた。

保護者回答の質問紙 (14 編) には，Selective Mutism Questionnaire (SMQ) (10/14 編)，FSSM の重症度尺度(1/14 編)があった。SMQ は学校，家庭，その他の社会的状況の発話場面の項目 （ *e.g.*, たいていの同輩と学校で話す，他の人がいても家で家族と話す，医師や歯科医と話す）における発話頻度を，4 件法で評価する尺度で， Bergman, Keller, Piacentini, and Bergman (2008) により，17 項目について，妥当性検証，標準化が行われている。FSSM 重症度尺度は，学校，家庭，その他の社会的状況における場面緘黙の症状の項目（ *e.g.*, あなたの子どもは全般的に同級生と話しますか，見知らぬ人が来ていても家で親しい家族と話しますか，医師と話しますか）についてどの程度あてはまるか，5 件法で評価する尺度である( Gensthaler *et al.*, 2020)。3–7 歳用 (41 項目)，6–11 歳用 (42 項目)，12–18 歳用 (41項目) の 3 種類がある。発話頻度を尋ねる項目の他，活動への参加頻度を尋ねる項目が含まれている。 Gensthaler *et al.* (2020) で妥当性検証が行われている。

教師回答の質問紙 (2 編) では，DortMus-Kita ( Starke & Subellok, 2018) (1/2 編) か School Speech Questionnaire( Bergman Piacentini, & McCracken, 2002) (1/2 編) が使用されていた。DortMus-Kita は，園や学校での子どもの発話行動，集団への参加を評価する 17 項目（ *e.g.*, 遊び場面で他の子どもに話しかける，先生に声をかけられても黙っている）についてどの程度あてはまるかを，5 件法で評価する質問紙である ( Starke, 2018)。 Starke and Subellok (2018) により，妥当性検証が行われている。School Speech Questionnaire は，SMQ ( Bergman, Keller, Wood, Piacentini, & McCracken, 2001) を基に作成されたものであり，学校での発話頻度を評価する 11 項目4件法の質問紙である ( Bergman *et al.*, 2002)。School Speech Questionnaire を使用したレビュー対象の研究 ( Bergman *et al.*, 2002) では，項目テスト相関の低かった 2 項目を除外した 9 項目が使用されていた。

面接を行って発話評価をした研究 ( Martinez *et al.*, 2015) では，教師を対象とした面接である Teacher Telephone Interview: Selective Mutism and Anxiety in the School Setting (TTI-SM; Tannock, Fung, & Manassis, 2003) の場面緘黙下位尺度を用いていた。TTI-SM の場面緘黙下位尺度は，場面緘黙の症状に関する 15 項目（ *e.g.*, たいていの同輩と学校で話す，先生からの問いに答える）について 4 件法で回答を求める電話面接である。発話頻度を尋ねる項目の他，非言語コミュニケーションの頻度，話していないことによる学校成績への影響を尋ねる項目が含まれている。 Martinez *et al.* (2015) により，妥当性検証が行われている。

観察により発話評価した研究 ( Edison *et al.*, 2011) では，実験室での場面緘黙児とその保護者の会話場面を録画し，子どもの発話と保護者の発話について観察者が評価した。観察場面は，保護者への教示によって4つのセグメント（ *e.g.*, 自由遊び場面）に分けられていた。発話は，1 つの主節，あるいは 1 つの主節に従属節や埋め込まれた節が加わったものを1単位とする T 単位に分割された。子どもの発話は，自発的な発話と保護者への応答に分類され，それぞれ，T 単位の総数が算出された。

**Table 3.** 場面緘黙診断確定以外を目的とした発話評価の手法.

研究	発話評価ツール	本人観察	本人質問紙	保護者質問紙	教師面接	教師質問紙
Gensthaler *et al.* (2020)	FSSM			✓		
Klein *et al.* (2019)	SMQ (2008)			✓		
Starke (2018)	オリジナル質問紙 DortMus-Kita			✓		✓
Martinez *et al.* (2015)	SMQ (2008) TTI-SM			✓	✓	
Edison *et al.* (2011)	観察（保護者との言語的相互作用）	✓				
Letamendi *et al.* (2008)	SMQ (2008)			✓		
Cohan *et al.* (2008)	SMQ (2001)			✓		
Bergman *et al.* (2008)	SMQ (2008)			✓		
Manassis *et al.* (2007)	SMQ (2001)			✓		
Chavira *et al.* (2007)	SMQ (2001)			✓		
Arie *et al.* (2007)	SMQ (2001)			✓		
Steinhausen *et al.* (2006)	オリジナル質問紙		✓			
McInnes *et al.* (2004)	SMQ (1999)			✓		
Manassis *et al.* (2003)	SMQ (1999)			✓		
Bergman *et al.* (2002)	School Speech Questionnaire					✓
Ford *et al.* (1998)	オリジナル質問紙		✓	✓		
Dummit *et al.* (1997)	オリジナル質問紙			✓		

## IV. 考察

### 1. 用いられた医学的診断基準

研究によって使用された診断基準が異なっており，結果の解釈には注意が必要である。用いられた診断基準には，ICD-9，ICD-10 ( World Health Organization, 1993;　中根・岡崎・藤原・中根・針間訳，2008)，DSM-III ( American Psychiatric Association, 1980)，DSM-III-R ( American Psychiatric Association, 1987)，DSM-IV ( American Psychiatric Association, 1994)，DSM-IV-TR ( American Psychiatric Association, 2000)，DSM-5 ( American Psychiatric Association, 2013) があった。ICD-9 では，精神疾患に起因しない広範かつ持続的な発話の拒否という記述が認められたものの ( National Center for Health Statistics, 1980, p.1091)，診断基準について詳細な情報は，著者らの調べる限りでは得られなかった。ICD-9 で “elective mutism: 選択的無言症” は確立された診断カテゴリーとして位置付けられておらず，“313: 児童期と青年期に特殊な感情障害” に含められていた。しかし，ICD-10 では “F94.0: elective mutism: 選択性緘黙”の診断が確立された上，“F94: 小児期および青年期に特異的に発症する社会機能の障害”の主要カテゴリーの下へ位置づけられたことは，ICD-9 と ICD-10 における場面緘黙の扱いが異なっていることを示している。DSM-IV，DSM-IV-TR，DSM-5 の診断基準は同一だが，これらの診断基準以外の診断基準では，社会的状況の種類や症状の持続期間など，それぞれ異なる点が存在している。DSM-III，DSM-III-R では，学校で話せないこと，多くの状況で話せないことが条件になっているが，他の診断基準では，話せない状況が学校である必要はなく，話せない状況が複数である必要もない。DSM-III，DSM-III-R では，症状の持続期間に関する明確な基準はないが，DSM-IV，DSM-IV-TR，DSM-5 では症状が1か月以上，ICD-10 では4週間以上持続することが診断基準に含まれている。言語表現及び言語理解について，ICD-10 では，標準化検査の得点が2標準偏差以内であることが基準として記載されているが，DSMはすべての版において明確な言語能力の基準はない。診断基準が異なる場合，研究で対象とする母集団が異なる可能性があるため，研究間で結果を単純に比較することはできない。個々の研究で用いた診断基準を考慮した上で，個々の研究の解釈や，研究間の比較をする必要がある。

診断基準によって，社会的状況の種類や症状の持続期間などに違いはあるが，話すことができる社会的状況と話すことができない社会的状況の両方があるという点はすべての診断基準に共通していた。どの診断基準を使用する場合であっても，場面緘黙診断確定のためには複数の社会的状況下での発話行動を評価する必要がある。

### 2. 場面緘黙の診断確定に用いられた手法

検査自体の妥当性を高めるためには，場面緘黙診断確定に使用する個々の面接や質問紙の標準化が必要である。診断確定に用いられた手法のうち，場面緘黙診断に関する妥当性が検証されていたのは FSSM のみだったが，FSSM を使用した研究は 60 編中 2 編だけだった。約 97% (58/60 編) の研究は，場面緘黙診断確定について，妥当性が検証されていない手法を用いていたか，診断確定方法についての十分な記載がなかった。標準化手続きを経た妥当性の高い評価手法でないと，妥当性の高い診断確定ができないため，標準化された手法を使用することが必要である。FSSM は原版のドイツ語版において，定型発達群，社交不安症群，内在化障害群（うつ病，限局性恐怖症，強迫症，全般不安症，分離不安症，パニック症特定不能の不安症，適応障害）との弁別能が支持されている ( Gensthaler *et al.*, 2020)。今後，他の多くの言語に翻訳され，妥当性検証が行われれば，幅広い地域で場面緘黙の診断確定が可能となるだろう。現在，日本語で使用可能な場面緘黙診断確定方法としては，Kiddie Schedule for Affective Disorders and Schizophrenia Present and Lifetime Version (K-SADS-PL) ( Kaufman *et al.*, 2016) が挙げられる。K-SADS-PL は DSM-IV に対応した版までは場面緘黙診断確定の項目は含まれていなかったが，DSM-5 に対応した版では場面緘黙の診断確定ができるようになっており，日本語版の妥当性が支持されている ( Nishiyama *et al.*, 2020)。 Nishiyama *et al.* (2020) はサンプルサイズが小さかった( *n*=4)ため，さらなる検証が必要だが，K-SADS-PL は日本語で使用可能な場面緘黙診断確定面接として有望かもしれない。

面接や質問紙の標準化に加えて，行動観察に基づく評価手法の開発も今後の課題である。質問紙や半構造化面接で扱われている内省的な報告は，実際の行動と乖離する可能性が，これまでの研究で問題点として指摘されている ( Baumeister, Vohs, & Funder, 2007)。質問紙や面接だけでなく，行動観察による発話評価を併せて行うことが妥当性の高い診断確定には必要だろう。レビュー対象の論文でも観察を行った研究は少ないながらも存在したが (6/60 編)，それぞれの研究が独自の観察を行っており，場面緘黙診断確定のために確立された観察法はなかった。また，複数の評価指標や情報に基づき，総合的に場面緘黙の診断を下すことを提唱しているガイドライン ( Dow, Sonies, Scheib, Moss, & Leonard, 1995) においても，複数の社会的状況下での発話行動の実測については言及されていない。今後の研究では，場面緘黙児・者の発話の観察を含めた評価手法の確立が望まれる。

### 3. 場面緘黙と他の障害との鑑別に用いられた手法

場面緘黙は他の不安症や神経発達症の併存が多く報告されており ( Kristensen, 2000; Steffenburg *et al.*, 2018)，妥当性の高い鑑別方法の確立が今後の課題である。先行研究においても，場面緘黙と他の障害との鑑別方法が確立されていないことが問題点として挙げられている ( Driessen *et al.*, 2020; Steffenburg *et al.*, 2018)。本研究の結果，場面緘黙群と他の障害群を比較する研究において，鑑別方法が確立されていないことが明らかになった。場面緘黙診断確定の面接や質問紙の妥当性検証においては，コミュニケーションの問題を示す場面緘黙以外の障害（ *e.g.*, 言語症，自閉スペクトラム症）との弁別能の検証が必要である。FSSM は場面緘黙と社交不安症や内在化障害群（うつ病，限局性恐怖症，強迫症，全般不安症，分離不安症，パニック症特定不能の不安症，適応障害）との鑑別については有用性が示唆されている ( Gensthaler *et al.*, 2020)。コミュニケーションの障害を示す点で共通している場面緘黙と自閉スペクトラム症等の神経発達症との鑑別についても検証されることが望ましい。場面緘黙以外にも，コミュニケーションの問題が症状として表れる障害（ *e.g.*, 言語症，自閉スペクトラム症）は存在するが，場面緘黙は状況によって発話行動が異なる点が他の障害と異なる特徴である。したがって，複数状況下での発話行動の評価をアセスメントに含めることで，他の障害との鑑別が可能になるかもしれない。

他の障害との鑑別が困難な理由として，場面緘黙の病因や行動特徴が場面緘黙児・者間で異なっている可能性や，介入効果が場面緘黙児・者間で共通していないことも影響しているかもしれない。よって，近年のレビュー論文 ( Rozenek, Orlof, Nowicka, Wilczyńska, & Waszkiewicz, 2020) でも指摘されているように，場面緘黙は同質性のある (homogeneity) 障害としてではなく，異質性のある (heterogeneity) 障害として概念化していくことが重要かもしれない。場面緘黙児・者内で行動特徴や心理要因を比較・検討した研究が数多く存在することからも，場面緘黙の異質性については検討する価値があるだろう。 Hayden (1980) では，病因や緘黙症状の意味によって場面緘黙児についてタイプ分けを行った結果，対象児は，保護者が支配的で保護者との結びつきが強い共生タイプ，自分の声を聞くことに恐怖を示すスピーチ恐怖タイプ，トラウマ体験をきっかけに発症した反応性タイプ，沈黙を武器として使用する受動的攻撃的タイプの4種に分類された。 Cohan *et al.* (2008) では，発話の欠如以外の症状を基に場面緘黙児 についてタイプ分けを行い，対象児は，不安と軽度の反抗を示すタイプ，不安とコミュニケーションの遅れを示すタイプ，不安のみを示すタイプの3種に分類された。 Diliberto and Kearney (2018) においても，発話の欠如以外の症状を基にタイプ分けが行われており，中程度の不安・攻撃性・不注意を示すタイプ，重度の不安と中程度の攻撃性・不注意を示すタイプ，軽度から中程度の不安と軽度の攻撃性・不注意を示すタイプの3種に分類された。このように，これまでにも複数のサブタイプに分類できる可能性が示されてきた。さらに，自閉スペクトラム症の症状を併存する場面緘黙児は，そうでない場面緘黙児と比べて，場面緘黙の発症が遅いことや( Steffenburg *et al.*, 2018)，同じ介入プログラムの効果は場面緘黙児内で異なっており，顕著な介入効果があるケースと症状が持続するケースがある ( Oerbeck, Stein, Pripp, & Kristensen, 2015; Oerbeck, Overgaard, Stein, Pripp, & Kristensen, 2018)。以上のことから，場面緘黙は異質性のある障害として再度概念化した上で，同質性 (homogeneity) と異質性 (heterogeneity) の両側面から，評価を進めていく必要があるのかもしれない。

場面緘黙の発症に関する遺伝的要因についても，今後さらなる研究が求められる。これまでの研究では，一般人口と比べ，場面緘黙児・者の家族や親戚では，場面緘黙や社交不安症 ( Black & Uhde, 1995)，精神疾患 ( Brix Andersson & Thomsen, 1998; Steinhausen & Adamek, 1997) を有する割合の高いことが報告されている。また，自閉スペクトラム症との関連が示唆されている contactin-associated protein-like 2-gene (CNTNAP2) の遺伝的多型の 1 種 (rs2710102) が場面緘黙に関連していることも報告されている ( Stein *et al.*, 2011)。

### 4. その他の発話評価手法

2008 年以降，SMQ，FSSM，DortMus-Kita，TTI-SM などの標準化された質問紙や面接が用いられるようになった。SMQ の日本語版である Selective Mutism Questionnaire-Revised (SMQ-R) ( かんもくネット，2011) は，現在，妥当性検証が進められており（ 角田，2021），日本でも標準化質問紙によって場面緘黙児の発話評価が可能になると期待される。しかし，小児・青年を対象とした標準化尺度や面接が作成されてきた一方で，成人を対象に含む標準化質問紙や面接は存在しないことが，本研究によって示された。SMQ は 3-11 歳 ( Bergman *et al.*, 2008)，FSSM は 3-18 歳 ( Gensthaler *et al.*, 2020)，DortMus-Kita は 3 歳0か月から 6 歳 11 か月( Starke & Subellok, 2018) を対象として標準化されている。SMQ を基に作成された TTI-SM は 6-11 歳を対象として標準化された ( Martinez *et al.*, 2015)。小児期や青年期だけでなく，成人期に場面緘黙症状を示すケースもあるため ( Ford, Sladeczek, Carlson, & Kratochwill, 1998; Walker & Tobbell, 2015)，成人場面緘黙者の発話を評価する標準化質問紙・面接の作成は今後の課題である。

質問紙や面接による測定だけでなく，複数状況下での実際の発話を定量的に測定する研究が更に必要である。複数状況下での観察を行い，実際の発話を定量的に測定した研究は，レビュー対象の論文のうち，1 編のみだった ( Edison *et al.*, 2011)。日常場面の行動を理解するためには，行動を直接観察する方法が適しているという指摘があり ( Baumeister *et al.*, 2007)，場面緘黙児・者の発話行動の特徴を明らかにするためには，行動観察が重要である。 Edison *et al.*(2011) では，実験室での複数種類の活動中の保護者との会話を観察し，場面緘黙児の自発的な発話と応答を定量的に評価した。質問紙調査によって場面緘黙児・者は場所，相手，活動によって発話頻度が異なると示されているが ( Dummit *et al.*, 1997)，場所，相手，活動による発話行動のを実験的検討は不足している。発話頻度以外の発話行動の特徴についても検討の余地がある。異なる場所での発話行動，異なる相手との発話行動， Edison *et al.* (2011) では検討されなかった異なる活動中の発話行動など，場面緘黙児・者の発話行動について，未検討の点が数多く残っている。今後は，発話行動に影響する複数の変数(場所，相手，活動など)について，場面緘黙児・者を対象とした実験的な研究を推進していくことが必要だろう。

## V. 結論

本研究は，調査・実験研究のみを対象としており，介入研究をレビューしていないという限界がある。また，事前にレビュープロトコルが準備されなかったという方法論的課題もあった。しかし，本研究によって，場面緘黙の調査・実験研究において発話評価手法が確立されていないという問題が明らかになった。今後の研究では，場面緘黙診断確定のため，また，場面緘黙と他の障害との鑑別のため，異なる社会的状況における発話評価手法を確立する必要がある。

### データ可用性

#### 基礎データ

本論文の研究結果の基礎となるデータはすべて本論文中に示されており，追加のソースデータは必要とされていない。

#### 報告のガイドライン

Open Science Framework: Measurement of speech in individuals with selective mutism: A systematic review. https://doi.org/10.17605/OSF.IO/ZS36Q ( Toma & Matsuda, 2022).

　・PRISMA_2020_checklist.pdf　(PRISMA2020チェックリスト)

　・PRISMA_2020_abstract_checklist.pdf　(PRISMA2020抄録チェックリスト)

データは， Creative Commons Zero “No rights reserved” data waiver (CC0 1.0 Public domain dedication) の条件の下で利用可能です。
