# Supplementary material for: Measurement of speech in individuals with selective mutism: A systematic review
Source: F1000Res. 2023 Jun 9;11:847. [Article in Japanese] Originally published 2022 Jul 28. [Version 4] doi: 10.12688/f1000research.113302.4 (PMC9958303; doi:10.12688/f1000research.113302.4)
Supplement: Peer review history

# Peer review: Reviewer response for version 4

**DOI:** 10.5256/f1000research.150463.r177398

**Published:** 2023-06-12

**Authors:** Shoji Okamura

**Affiliations:** 1 Education for Children with Developmental Disabilities, Hyogo University of Teacher Education, Hyogo, Japan

すべて確認できました。適切に対応いただき、ありがとうございました。

（該当する場合は要回答）統計分析および解釈は適切ですか。

Yes

システマティックレビューの根拠と目的は明確に示されていますか。

Yes

方法と分析について第三者による再現が可能となるよう十分な詳細が提示されていますか。

Yes

結論はレビューの結果により妥当な裏付けを得ていますか。

Partly

Reviewer Expertise:

発達障害臨床心理学　応用行動分析学　特別支援教育

I confirm that I have read this submission and believe that I have an appropriate level of expertise to confirm that it is of an acceptable scientific standard.

# Peer review: Reviewer response for version 3

**DOI:** 10.5256/f1000research.147031.r173407

**Published:** 2023-05-31

**Authors:** Shoji Okamura

**Affiliations:** 1 Education for Children with Developmental Disabilities, Hyogo University of Teacher Education, Hyogo, Japan

これまでのコメントに基づき、丁寧に修正いただきありがとうございました。今後の研究の大きな手掛かりになる有益な論文であると考えます。以下、ご確認ください。

 ・P10L1　23編でしょうか。

 ・Table 2　「場面緘黙診断確定を目的として開発された測定法の種類」にチェックがある論文とない論文があります（例えば、K-SADS-PLは面接で実施されると思われますが、チェックがついていません）。「開発された測定法」ということなので、方法の詳細が明記されているか否かで判断されているかと思われますが、P10L5に簡潔にチェックの基準を追記してください。

（該当する場合は要回答）統計分析および解釈は適切ですか。

Yes

システマティックレビューの根拠と目的は明確に示されていますか。

Yes

方法と分析について第三者による再現が可能となるよう十分な詳細が提示されていますか。

Yes

結論はレビューの結果により妥当な裏付けを得ていますか。

Partly

Reviewer Expertise:

発達障害臨床心理学　応用行動分析学　特別支援教育

I confirm that I have read this submission and believe that I have an appropriate level of expertise to confirm that it is of an acceptable scientific standard.

# Response

**Published:** 2023-06-02

**Authors:** 友里亜 藤間

**Affiliations:** 筑波大学大学院, Japan

岡村先生

 この度は，査読のために貴重なお時間を割いてくださり，誠にありがとうございました。

 いただいたコメントについて確認いたしました。

 ・P10L1

 診断確定の手法として，複数の方法の併用や医療記録によって総合的に判断した研究の数を再確認したところ，24編でした（Klein *et al*., 2019; Steffenburg *et al*., 2018; Muchnick *et al*., 2013; Levin-Decanini *et al*., 2013; Klein *et al*., 2013; Alyanak *et al*., 2013; Young *et al*., 2012; Henkin *et al*., 2010; Bergman *et al*., 2008; Chavira *et al*., 2007; Arie *et al*., 2007; Kristensen & Oerbeck, 2006; Cunningham *et al*., 2006; McInnes *et al*., 2004; Cunningham *et al*., 2004; Bar-Haim *et al*., 2004; Manassis *et al*., 2003; Kristensen, 2002; Kristensen & Torgersen, 2001; Kristensen, 2001; Kristensen, 2000; Brix Andersson & Hove Thomsen, 1998; Dummint *et al*., 1997; Wilkins, 1985）。

 ・Table 2

 測定法の種類に関しては，場面緘黙診断確定を目的として開発された測定法，または，場面緘黙診断モジュールが含まれている測定法のみ，チェックをつけました。レビュー対象の論文の中では，場面緘黙診断確定に使用されたと記載されていても，場面緘黙診断確定を目的として開発されたわけではない測定法もありました。このような測定法については，「場面緘黙の特定を目的として開発された測定法の種類」の列にはチェックをつけませんでした。K-SADS-PLはDSM-5に対応した版では，場面緘黙診断モジュールが含まれておりますが，レビュー対象の論文で使用されていたK-SADS-PLはDSM-Ⅲ-R，DSM-Ⅳに対応した版であり，場面緘黙診断モジュールが含まれていなかったため，「場面緘黙の特定を目的として開発された測定法の種類」の列にはチェックをつけておりません。この点について，本文中に加筆いたしました。

 該当箇所

 Ⅲ．結果　3．場面緘黙の診断確定に用いられた手法　第1段落

 …場面緘黙の診断を確定する目的で開発された測定法を使用した研究と，他の目的で開発された測定法（ *e.g., *知能検査，臨床面接）を場面緘黙診断確定のために使用した研究があった。場面緘黙の診断を確定する目的で開発された測定法は Table 2 中に太字で示した。SMQ は場面緘黙の重症度を評価する尺度だが，診断確定手続きに使用したと記載された研究があり，診断確定の列にも含まれている。診断確定を目的とした測定法ではないため，診断確定の列には細字で示した。測定法の種類については，場面緘黙診断確定を目的として開発された測定法の種類のみを示した。場面緘黙診断確定に使用したと論文中に記載されていても，場面緘黙診断確定を目的として開発されたわけではない測定法については，「場面緘黙の特定を目的として開発された測定法の種類」の列にチェックを記入していない。

# Peer review: Reviewer response for version 3

**DOI:** 10.5256/f1000research.147031.r173405

**Published:** 2023-05-16

**Authors:** Shin-ichi Ishikawa

**Affiliations:** 1 Faculty of Psychology, Doshisha University, Kyoto, Japan

考察に関するコメントに適切に対応していただいているのを確認いたしました。これ以上のコメントはございません。修正いただきありがとうございました。

（該当する場合は要回答）統計分析および解釈は適切ですか。

Not applicable

システマティックレビューの根拠と目的は明確に示されていますか。

Yes

方法と分析について第三者による再現が可能となるよう十分な詳細が提示されていますか。

Partly

結論はレビューの結果により妥当な裏付けを得ていますか。

Yes

Reviewer Expertise:

臨床児童心理学

I confirm that I have read this submission and believe that I have an appropriate level of expertise to confirm that it is of an acceptable scientific standard.

# Response

**Published:** 2023-06-02

**Authors:** 友里亜 藤間

**Affiliations:** 筑波大学大学院, Japan

石川先生

 この度は，査読のために貴重なお時間を割いてくださり，誠にありがとうございました。

# Peer review: Reviewer response for version 2

**DOI:** 10.5256/f1000research.140745.r157267

**Published:** 2023-02-24

**Authors:** Yoshihiro Kanai

**Affiliations:** 1 Faculty of Liberal Arts, Tohoku Gakuin University, Miyagi, Japan

コメントに従って適切に修正されています。行動観察に基づく評価手法を行なっていた研究において，どのような独自の観察を行っていたのかが加筆され，現状がよく理解できました。また，機能的アセスメントの必要性に関する考察部分の加筆についても，後続刺激とともに先行刺激の制御の視点も述べられ，読者にとって有益な情報になりました。

（該当する場合は要回答）統計分析および解釈は適切ですか。

Yes

システマティックレビューの根拠と目的は明確に示されていますか。

Yes

方法と分析について第三者による再現が可能となるよう十分な詳細が提示されていますか。

Yes

結論はレビューの結果により妥当な裏付けを得ていますか。

Yes

Reviewer Expertise:

NA

I confirm that I have read this submission and believe that I have an appropriate level of expertise to confirm that it is of an acceptable scientific standard.

# Response

**Published:** 2023-04-14

**Authors:** 友里亜 藤間

**Affiliations:** 筑波大学大学院, Japan

金井先生

 この度はご多忙の中，査読をしてくださり，誠にありがとうございました。

# Peer review: Reviewer response for version 2

**DOI:** 10.5256/f1000research.140745.r157265

**Published:** 2023-01-10

**Authors:** Shoji Okamura

**Affiliations:** 1 Education for Children with Developmental Disabilities, Hyogo University of Teacher Education, Hyogo, Japan

修正いただきありがとうございました。より興味深い論文になり大変価値のある研究であると考えます。以下、ご検討いただければと思います。

 ・「２　場面緘黙の診断確定に用いられた手法」では、観察を行った研究が6編と少なく、一貫して話していない状況の観察が特に不足しており、観察の手法の確立が求められていると考察しています。一方、「４　その他の発話評価手法」でも観察により発話評価した研究は1編のみであることを指摘し、行動観察に求められる内容について考察しています。ただ行動観察の内容や手法については目的（診断や鑑別か介入のためのアセスメントか）によって変わってくると考えられます（診断確定に用いられた手法において、観察を行った研究が少ないのは少ないなりの理由もあるかと想定されます）。目的に応じて行動観察が違ってくる点について明記すべきだと考えます。

 ・異質性について機能の観点から分析していることは興味深いです。しかしながら、環境要因に加えて、後段で語られる遺伝的要因等を含めて、場面緘黙を異質性のある障害として概念化していくことが求められるのではないでしょうか。

 ・P16「3．場面緘黙と他の障害との鑑別に用いられた手法」において、ASD等との神経発達症との鑑別については未検証であると指摘していますが、場面緘黙とASDの併存群と場面緘黙群を比較した唯一の研究結果をもとに何らかの考察はできないでしょうか。

 ＜その他＞

 ・P6「3．場面緘黙の診断確定に用いられた手法」で妥当性検証の有無の確認に関する手続きですが、Ⅱ方法の４で記述してはいかがでしょうか。

 ・P13 「5．その他の発話評価手法」　診断確定以外の目的だけでなく、他の障害との鑑別以外の目的も含まれます。

 ・Table 2　Wilkinson (1985) の診断基準は「-」？

（該当する場合は要回答）統計分析および解釈は適切ですか。

Yes

システマティックレビューの根拠と目的は明確に示されていますか。

Yes

方法と分析について第三者による再現が可能となるよう十分な詳細が提示されていますか。

Yes

結論はレビューの結果により妥当な裏付けを得ていますか。

Partly

Reviewer Expertise:

特別支援教育　応用行動分析学

I confirm that I have read this submission and believe that I have an appropriate level of expertise to confirm that it is of an acceptable scientific standard, however I have significant reservations, as outlined above.

# Response

**Published:** 2023-04-14

**Authors:** 友里亜 藤間

**Affiliations:** 筑波大学大学院, Japan

岡村先生

 この度はご多忙の中，査読をしてくださり，誠にありがとうございます。

 いただいたコメントをもとに以下のように加筆・修正を行いました。

 ・求められる行動観察が目的によって異なるという点に関して

 ご指摘の通り，求められる行動観察は目的によって異なると考えられます。診断確定のためには，特定の状況で話していることと，他の状況で話していないことの確認が必要であり，診断確定の手法は，統一されたアセスメント手法が用いられることが重要だと考えられます。一方で，場面緘黙の重症度を評価するなど従属変数として発話行動を測定する際には，様々な状況において話しているか否かだけでなく，発話行動がどの程度表出されているか連続量で評価する必要があり，また，個別の症状・行動傾向に応じた多様な発話評価が必要になると考えられます。この点について，考察2「場面緘黙の診断確定に用いられた手法」，4「その他の発話評価手法」に加筆いたしました。

 該当箇所

 Ⅳ．考察　2．場面緘黙の診断確定に用いられた方法　第2段落

 …場面緘黙診断確定のための観察としては，特定の状況（ *e.g.*, 学校）で話していないことと，他の状況（ *e.g.*, 家庭）で話していることを確認する必要がある。

 Ⅳ．考察　4．その他の発話評価手法　第2段落

 …場面緘黙の重症度等を評価するためには，様々な状況において話しているか否かだけでなく，発話行動がどの程度表出されているか評価する必要がある。例えば，実験的に場所，相手，活動などを操作し，発話量等を定性的にではなく，定量的に評価することが考えられる。

 ・遺伝的要因を含めて場面緘黙を異質性のある障害として概念化する必要があるのではないかという点に関して

 ご指摘の通り，遺伝的側面についても異質性のある障害として考える必要があると考えています。以前の版では，意図が不明確だったため，遺伝的異質性について，考察３「場面緘黙と他の障害との鑑別に用いられた手法」の箇所を加筆・修正いたしました。

 該当箇所

 Ⅳ．考察　3．場面緘黙と他の障害との鑑別に用いられた手法　第3段落

 場面緘黙に関する遺伝的要因の解明も，異質性のある障害としての場面緘黙の概念化に寄与すると考えられる。自閉スペクトラム症との関連も示唆されている contactin-associated protein-like 2-gene (CNTNAP2) の遺伝的多型の 1 種 (rs2710102) が場面緘黙に関連していたことを報告した研究 ( Stein et al., 2011)は存在するものの，場面緘黙と複数の神経発達症・精神疾患の間でゲノムデータを比較した研究はこれまでにない。また，一般人口と比べ，場面緘黙児・者の家族や親戚では，場面緘黙や社交不安症 ( Black & Uhde, 1995)，精神疾患 ( Brix Andersson & Hove Thomsen, 1998; Steinhausen & Adamek, 1997) を有する割合の高いことも報告されているが，疫学的研究に限定されており，脳イメージングデータやゲノムデータとの関連を示した研究はない。場面緘黙には多様な遺伝的要因が関連している可能性が考えられることから，遺伝的同質性及び異質性を明らかにするためにも，その生物学的基盤を検討する研究の増加が望まれる。

 ・場面緘黙とASDの併存群と場面緘黙群を比較した研究をもとに，場面緘黙とASDの鑑別について考察できないかという点に関して

 場面緘黙とASDは類似しており，その鑑別が課題となっていることから，場面緘黙とASDの弁別能について，検証の余地があると思います。しかし，対人コミュニケーションの困難という行動特徴の類似性のため，単一の質問紙のみによって場面緘黙とASDを正確に鑑別することは困難である可能性が考えられます。検証の結果，単一の質問紙では鑑別ができないという結果が得られたならば，他の手法との併用が望ましいと考えられます。場面緘黙とASDの併存群と場面緘黙群を比較した研究では場面緘黙児を対象とした面接，保護者を対象とした面接，保護者回答の質問紙，教師回答の質問紙が用いられていました。この研究のように，複数のインフォーマントから情報を収集したり，複数状況での行動観察を行ったりすることがアセスメント手法として考えられると思います。この点について，考察3「場面緘黙とほかの障害の鑑別に用いられた手法」に加筆・修正を行いました。

 該当箇所

 Ⅳ．考察　3．場面緘黙と他の障害との鑑別に用いられた手法　第3段落

 …しかし，コミュニケーションの障害を示す点で共通している場面緘黙と自閉スペクトラム症等の神経発達症との鑑別については未検証だった。場面緘黙と自閉スペクトラム症は，その鑑別が課題となっており，弁別能について，検証の余地がある。しかし，対人コミュニケーションの困難という行動特徴の類似性のため，単一の質問紙のみによって場面緘黙と自閉スペクトラム症を正確に鑑別することは困難である可能性がある。検証の結果，単一の質問紙では鑑別ができないという結果が得られた場合，他の手法との併用が考えられる。場面緘黙と自閉スペクトラム症の併存群と場面緘黙群を比較した研究（Steffenburg et al., 2018）では場面緘黙児を対象とした面接，保護者を対象とした面接，保護者回答の質問紙，教師回答の質問紙が用いられていた。Steffenburg *et al*（2018）のように複数のインフォーマントから情報を収集したり，複数状況での行動観察を行ったりすることがアセスメント手法として考えられる。

 ・結果「3．場面緘黙の診断確定に用いられた手法」に記載していた妥当性検証の有無の確認に関する手続きについて，方法４に移動しました。

 ・「その他の発話評価手法」に関して

 「その他の発話評価手法」は診断確定及び他の障害との鑑別を目的とした評価以外に行われた評価の手法でした。本文とTable3のタイトルを修正しました。

 該当箇所

 Abstract

 …17編の研究は，診断確定及び他の障害との鑑別以外の目的で発話を測定していた。

 Ⅱ．方法　4．情報の抽出と統合

 …(4) その他の発話評価手法に関しては，診断確定及び他の障害との鑑別以外の目的で発話評価を行った研究を対象に，用いられた測定法の種類ごとに用いられた研究数，用いられた尺度について調べた。

 Ⅲ．結果　5．どの他の発話評価手法　第1段落

 　診断確定及び他の障害との鑑別以外の目的で発話評価を行った研究は 17/60 編あった。診断確定及び他の障害との鑑別以外の目的で用いられた発話評価の手法を出版年の新しい順に示した( Table 3)。

 ・Wilkinson (1985)の診断基準は「-」でした。追記いたしました。

# Peer review: Reviewer response for version 2

**DOI:** 10.5256/f1000research.140745.r157266

**Published:** 2022-12-12

**Authors:** Shin-ichi Ishikawa

**Affiliations:** 1 Faculty of Psychology, Doshisha University, Kyoto, Japan

　ご丁寧に改稿いただき，ありがとうございました。前回のコメントで触れさせていただきました点については，おおよそクリアになったかと思います。今回のコメントを見せていただき，改めて前回のコメントを通じて感じていた疑問点があります。以下の点をご検討いただければと思います。

 　本レビュー論文で扱っているのは，実証研究としての診断であり，診療報酬等とは関連していないものと思います。日本語においては，そのあたりがややこしいので，脚注にでも明記しても良いかもしれません。あるいは，実証研究における診断という形で限定しても良いと思います。

 　それは前提として，本研究では，ケースコントロールスタディを中心にレビューしているために，診断という変数は独立変数になるのだと思いますが，介入研究では主診断が外れるという形での従属変数になりえるかと思います（異質性の中において，こちらが特性でない限り）。そう考えると，「場面緘黙の診断確定に用いられた手法」と「その他の発話評価手法」との弁別は，本研究では問題ありませんが，場面緘黙全体の研究に拡張してしまうと少し矛盾が生じるかと思います。

 　その点は本質ではなく，操作的なものであり，おそらくここでの問題点は，診断の有無を確定する際のアセスメント手法が確定してないということかと思います。ASDや不安症のように，ゴールドスタンダードと呼べる方法を確立することが必要であること，それとは別に個別支援に有用な情報を得るためのアセスメントも必要不可欠であるということを論じたいのだと愚考しております。もしその理解でよろしければ，「場面緘黙の診断確定に用いられた手法」と「その他の発話評価手法」が，方法や内容が研究によって混在している現状が問題であることをもう少し明確に考察の中で指摘していただければ幸いです。

 　その一方で，行動観察が「場面緘黙の診断確定に用いられた手法」と「その他の発話評価手法」の両方に必要不可欠であるのか，という点については，利便性と頑健性の観点から議論が残るかと思います。いずれにしても，両者の目的においては，詳細さや定量化という観点から目的が異なるように思いますので，そのいずれにも必要であるということであれば，その違いを明確に論じていただけるとわかりやすいように思います。たとえば，診断においては短い家族が録画した動画等で実際に発話があるという事実（0，1）を確認することが重要であるのに対して，発話量などを測定する際には，ある程度固定された場面において，定められた時間観察が必要になり，評定にもアンカーポイント（例えば，のどが震えている，無声音が出る）などが導入される必要があるように思いました。両者を単に行動観察であると論じてしまうと，そのあたりが少しわかりにくいかなと思った次第です。　

 　いずれに観点も現状の原稿でも推察はできるのですが，考察内でも結果を網羅的に述べている分量が多いので，そのあたりの論点が薄まっているように感じられます。一度ご検討いただければ幸いです。

 以上です。

（該当する場合は要回答）統計分析および解釈は適切ですか。

Not applicable

システマティックレビューの根拠と目的は明確に示されていますか。

Yes

方法と分析について第三者による再現が可能となるよう十分な詳細が提示されていますか。

Partly

結論はレビューの結果により妥当な裏付けを得ていますか。

Yes

Reviewer Expertise:

臨床児童心理学

I confirm that I have read this submission and believe that I have an appropriate level of expertise to confirm that it is of an acceptable scientific standard, however I have significant reservations, as outlined above.

# Response

**Published:** 2023-04-14

**Authors:** 友里亜 藤間

**Affiliations:** 筑波大学大学院, Japan

石川先生

 この度はご多忙の中，査読をしてくださり，誠にありがとうございます。

 いただいたコメントをもとに以下のように加筆・修正を行いました。

 ・本論文で扱っているのは実証研究としての診断であるという点に関して

 脚注に，以下の文章を追記しました。「本研究においては，日本国内の診療報酬等に関連する診断ではなく，実証研究における診断という意味で，「診断」の用語を用いる。」

 ・「場面緘黙の診断確定に用いられた手法」と「その他の発話評価手法」という弁別に関して

 ご指摘の通り，「場面緘黙の診断確定に用いられた手法」が独立変数，「その他の発話評価手法」が従属変数という分け方は，介入研究を含む場面緘黙全体の研究には当てはめられないと思います。本研究のレビューでは，介入研究のように診断が従属変数となる研究を対象に含めなかったため，このような分け方をしました。

 ・ゴールドスタンダードと呼べる診断確定の方法，個別支援のためのアセスメントの両方が必要だという点に関して

 コメントいただいた通り，診断確定の方法，個別支援に有用な情報を得るためのアセスメントの両方が必要だと考えております。診断確定に関しては，手法を確立し，多くの研究者が同じアセスメント手法を用いることが重要だと考えています。その一方で，個別支援においては，個別の症状に応じた発話を評価するために，多様なアセスメント手法を用いることも重要だと考えられます。この点に関して考察に加筆いたしました。

 該当箇所

 Ⅳ．考察　2．場面緘黙の診断確定に用いられた方法　第1段落

 　検査自体の妥当性を高めるためには，場面緘黙診断確定に使用する個々の面接や質問紙の標準化が必要である。個別支援においては，個人の症状に応じた発話評価のためのアセスメント手法が必要となる一方で，研究における診断確定の手続きでは，研究間で共通したアセスメント手法が用いられることが求められる。

 ・診断確定の目的でもその他の目的でも行動観察が必要なのかという点に関して

 場面緘黙の主症状は特定状況における発話の欠如であるため，診断確定のための評価と診断確定以外を目的とした評価の両方に行動観察は必要だと考えております。しかし，ご指摘の通り，目的によって必要な評価内容は異なると考えられるため，どのような行動観察が求められるのか，考察2「場面緘黙の診断確定に用いられた手法」，4「その他の発話評価手法」のところにそれぞれ，説明を加えました。

 該当箇所

 Ⅳ．考察　2．場面緘黙の診断確定に用いられた方法　第2段落

 …場面緘黙診断確定のための観察としては，特定の状況（ *e.g.*, 学校）で話していないことと，他の状況（ *e.g.*, 家庭）で話していることを確認する必要がある。

 Ⅳ．考察　4．その他の発話評価手法　第2段落

 …場面緘黙の重症度等を評価するためには，様々な状況において話しているか否かだけでなく，発話行動がどの程度表出されているか評価する必要がある。例えば，実験的に場所，相手，活動などを操作し，発話量等を定性的にではなく，定量的に評価することが考えられる。

 ・考察内でも結果を述べている分量が多く，論点が薄まっているという点に関して

 考察内で結果について述べている部分を見直し，削除しても問題ないと考えられる部分を削除しました。

 該当箇所（""内を削除）

 Ⅳ．考察　2．場面緘黙の診断確定に用いられた方法　第2段落

 …複数状況での発話行動を定量的に評価し・比較し，状況によって発話行動が顕著に異なることを確認できるような観察手続きやチェック項目が確立されると，場面緘黙児・者を対象とした研究の参加者を選定する際に有用である。" *これまでの調査・実験研究では，観察を含む診断確定を行った研究は少なかった（6編）が，"*手法の確立によって，観察を実施する研究の増加が期待される。

# Peer review: Reviewer response for version 1

**DOI:** 10.5256/f1000research.125033.r145714

**Published:** 2022-08-29

**Authors:** Shoji Okamura

**Affiliations:** 1 Education for Children with Developmental Disabilities, Hyogo University of Teacher Education, Hyogo, Japan

場面緘黙の鑑別の困難性を背景とした介入の困難性は以前から指摘されており、診断方法を含めた発話評価を整理した価値ある研究です。結果を踏まえて発話評価手法に関するさらなる考察を深めていただければと考えます。

 ・P12L50　観察を行った研究は6編存在したとあるが、結果に示されていないため、「独自の観察の詳細」が不明です（Table2でもどの論文が対応するかが分かりません）。これらの内容を示しながら、どのような「発話の観察を含めた評価手法」が望まれるかについて考察してください。また、観察を行った研究がそもそも少ないことについてどう考察されますか（その他の発話評価方法も含め）。

 ・P13　本研究の結果、場面緘黙群と他の障害群との鑑別方法が確立されていないことが明らかになった、とあります。しかしながら、P8の結果をみる限り、少なくとも不安症群との鑑別（FSSMの有用性を含め）についてはある一定の方法が示されているとも読み取れます。確立されていないものの、何がどこまで示されているかを丁寧に記述する必要があると考えます。

 ・コミュニケーションの問題が症状として表れる障害との鑑別では、複数状況下での発話行動の評価により場面緘黙の診断は可能となると考えます。併存しているか否かを判断する場合には、どのようなアセスメントが求められるかを検討する必要があると考えます。

 ・同質性と異質性についてタイプ分けの先行研究を示しながら論じ、両側面から評価を進める必要があるとしています。読者がタイプ分けに関する先行研究を挙げる意味を理解するためにも、同質性と異質性の内容をより具体的に論じる必要があると考えます。特異的気質、環境要因、発達障害といった素因と遺伝的要因の相互作用としての障害として捉え、それらと関連させながら、複数状況下での発話行動の評価を行う必要があると指摘したいのでしょうか。

 ・P13「４　その他の発話評価手法」　複数の状況下での発話行動の定量的な測定、場所、相手、活動による発話行動、および頻度以外の発話行動の特徴に関する検討の必要性を指摘しています。診断確定以外の目的で発話評価を行った17編の研究をもとに考察しているため、介入にあたって必要な検討であると思われます。しかしながら、発話評価手法を検討するにあたって、診断確定を目的とする場合と介入のためのアセスメントや介入のための知見を示すことを目的とする場合で何がどう違うべきなのでしょうか。介入にあたっては、発話行動だけでなく、非言語行動を含めた行動全般の特徴を査定していくことが求められます。本レビューでは調査・実験研究を対象とはしていますが、目的に応じた評価手法について考察を展開していくことが望まれます。

 ＜その他＞

 ・「場面緘黙児・者の診断方法を含めた発話評価」など、題目（主題）を再考してください。

 ・2段階目のスクリーニングで「著者間で判断易相違があった場合」には協議を行ったとあります。しかしながら、結果の選定結果では、主に調査・実験研究以外の研究が除外されており、相違が起こり得にくいと読み取れます。判断に相違があった場合の適格基準について明記してください。

 ・医学的診断基準が明記されていた44編と診断確定に用いられた測定法について記載していた44編は一致していません。測定法が記載しているものの、使用した医学的診断基準が明記されていない論文があることは記述すべき事項かと考えます。

 ・Table 1でICD-9の記述をしてください。

 ・Table 2では44編中、36編が示されています。面接22編中、20編のみがチェックされています。理由を付記してください。

 ・P7L39　FSSMの引用文献は以下でしょうか

 Gensthaler. A, Dieter. J, Raisig. S et al (2018) Evaluation of a novel parent-rated scale for selective mutism. Assessment.

 ・P7L52　群間比較を行った研究が27編あります。内訳として、社交不安症10編、複数の不安症10編、複数の疾患6編、障害・疾患が不明2編だけで28編になります。記述の仕方を再考してください。複数の疾患6編についてはその後の記述がありませんが、特記する必要はないのでしょうか。

 ・Table 2のADIS-Ⅳは太字でしょうか。

 ・診断確定以外の目的での発話評価手法で用いられた質問紙では妥当性検証が行われているものが多いですが、診断目的ではFSSMのみです。どう考察しますか。

 ・P13L8　「発話行動のを実験的検討」→「を」削除

（該当する場合は要回答）統計分析および解釈は適切ですか。

Yes

システマティックレビューの根拠と目的は明確に示されていますか。

Yes

方法と分析について第三者による再現が可能となるよう十分な詳細が提示されていますか。

Yes

結論はレビューの結果により妥当な裏付けを得ていますか。

Partly

Reviewer Expertise:

特別支援教育　応用行動分析学

I confirm that I have read this submission and believe that I have an appropriate level of expertise to confirm that it is of an acceptable scientific standard, however I have significant reservations, as outlined above.

# Response

**Published:** 2022-11-04

**Authors:** 友里亜 藤間

**Affiliations:** 筑波大学大学院, Japan

岡村先生

 査読コメントを下さり誠にありがとうございます。

 以下のように加筆・修正を行いました。

 ・P12L50　観察を行った研究に関して

 Table2に観察実施の有無の列を追加し，どの研究が観察を実施したのか明らかにしました。行動観察を実施していた6編の研究のうち5編で家庭場面の録画・録音によって，家庭で話していることが確認されていました。そのうち１編は，家庭場面の録画・録音に加えて，実験室場面の発話行動も確認されました。観察が行われた６編のうち1編は，どのような観察を実施したのか，詳細な記述はありませんでした。診断確定のために観察を実施した６編の研究について結果3に加筆いたしました。

 　レビューの結果，家庭以外の場面の観察が特に不足していました。場面緘黙児の多くは学校で緘黙症状を示すため，学校場面の観察が有効だと考えられますが，手続き上の困難により，学校での観察が行われにくいのではないかと考え，考察2に加筆いたしました。

 　その他の発話評価方法として，複数状況の観察を行った研究は，Edison *et al*. (2011) 1編でした。Edison *et al*. (2011) は，複数の実験条件を設定し，場面緘黙児と保護者の会話を観察していました。異なる場所での発話行動，異なる相手との発話行動，Edison *et al*.（2011）では検討されなかった異なる活動中の発話行動など，場面緘黙児・者の発話行動について，さらに実験的検討が必要だと考えています。この点について，考察4に記載しました。

 該当箇所

 Ⅲ．結果　3. 場面緘黙の診断確定に用いられた手法　第1段落

 　診断基準，診断を確定するために用いられたと論文中で記載された測定法，測定法の種類，観察実施の有無について，出版年の新しい順に示した（Table 2）。

 Ⅲ．結果　3. 場面緘黙の診断確定に用いられた手法　第1段落

 …場面緘黙診断確定手法として確立された観察手続きはなかったが，発話行動の観察は診断確定のために重要であると考え，観察実施の有無と観察の内容についても確認した。

 Ⅲ．結果　3. 場面緘黙の診断確定に用いられた手法　第6段落

 　各研究独自の観察が診断確定に含まれていた研究が6/60編存在した（Arie *et al*., 2007; Bar-Haim *et al*., 2004; Henkin, Feinholz, Arie, & Bar-Haim, 2010; Kristensen, 2000; Kristensen & Oerbeck, 2006; Muchnik *et al*., 2013）。6編すべての研究において，観察と観察以外の手法を組み合わせて診断確定が行われていた。Bar-Haim *et al*.（2004），Henkin *et al*.（2010）， Muchnik *et al*.（2013）で実施された観察では，家庭場面の録画・録音によって，話す能力のあることが確認された。Kristensen（2000）では，家庭場面の録音によって，言語スキルが評価された。Arie *et al*.（2007）では，家庭場面の録画・録音による話す能力の確認に加え，実験室での保護者および見知らぬ実験者との相互作用中の発話行動も観察された。Kristensen and Oerbeck（2006）は，診断確定の手続きに直接観察が含まれていたと記載されていたが，どのような場面の観察を実施したのかについて詳細な記載はなかった。

 Table 2 最右列

 Ⅳ．考察　2. 場面緘黙の診断確定に用いられた手法　第2段落

 …面接や質問紙の標準化に加えて，行動観察に基づく評価手法の開発も今後の課題である。複数の評価指標や情報に基づき，総合的に場面緘黙の診断を下すことを提唱しているガイドライン（Dow, Sonies, Scheib, Moss, & Leonard, 1995）においても，複数の社会的状況下での発話行動の実測については言及されていない。しかし，質問紙や半構造化面接で扱われている内省的な報告は，実際の行動と乖離する可能性が，これまでの研究で問題点として指摘されている（Baumeister,  Vohs, & Funder, 2007）。質問紙や面接だけでなく，行動観察による発話評価を併せて行うことが妥当性の高い診断確定には必要だろう。今後の研究では，場面緘黙児・者の発話の観察を含めた評価手法の確立が望まれる。レビュー対象の論文でも観察を行った研究は少ないながらも存在しており（6/60編），5/6編において家庭場面で問題なく話していることが確認されていた。1/6編の研究では，家庭場面の観察に加えて，家庭以外の場面の観察も実施された。ただし，それぞれの研究が独自の観察を行っており，場面緘黙診断確定のために確立された観察法はなかった。また，これまでの調査・実験研究では，一貫して話していない状況の観察が特に不足していた。場面緘黙児の多くは学校で話すことができないため（Rodrigues Pereira *et al*., 2021），学校場面の録画を提出するよう求めることが有用だと考えられるが，普段の授業時間や休み時間など，児童・生徒が多数いる状況を録画することについて同意を得るのは手続き上の困難があると推察される。場面緘黙当事者と，特定の話すことができない相手（例えば，担任教員）の同意を得て，同意が得られた人々だけの状況を設定してもらい，その状況を録画し提出するよう依頼するのが現実的かもしれない。このように設定された状況は，一貫して話していない普段の状況とは異なってしまうが，話していない状態は確認できる。…Arie *et al*.（2007）で実施されていたように複数状況での観察を行うことが望ましいと考えられる。複数状況での発話行動を定量的に評価し・比較し，状況によって発話行動が顕著に異なることを確認できるような観察手続きやチェック項目が確立されると，場面緘黙児・者を対象とした研究の参加者を選定する際に有用である。これまでの調査・実験研究では，観察を含む診断確定を行った研究は少なかった（6編）が，手法の確立によって，観察を実施する研究の増加が期待される。

 Ⅳ．考察　4. その他の発話評価手法　第2段落

 　質問紙や面接による測定だけでなく，複数状況下での実際の発話を定量的に測定する研究が更に必要である。質問紙や面接を用いて複数状況下での発話評価を行った研究はあったものの，複数状況下での観察を行い，実際の発話を定量的に測定した研究は，レビュー対象の論文のうち，1編のみだった（Edison *et al*., 2011）。日常場面の行動を理解するためには，行動を直接観察する方法が適しているという指摘があり（Baumeister *et al*., 2007），場面緘黙児・者の発話行動の特徴を明らかにするためには，行動観察が重要である。Edison *et al*.（2011）では，実験室での複数種類の活動中の保護者との会話を観察し，場面緘黙児の自発的な発話と応答を定量的に評価した。質問紙調査によって場面緘黙児・者は場所，相手，活動によって発話頻度が異なると示されているが（Dummit *et al*., 1997），場所，相手，活動による発話行動の実験的検討は不足している。また，発話頻度以外の発話行動の特徴についても検討の余地がある。異なる場所での発話行動，異なる相手との発話行動，Edison *et al*.（2011）では検討されなかった異なる活動中の発話行動など，場面緘黙児・者の発話行動について，未検討の点が数多く残っている。今後は，発話行動に影響する複数の変数（場所，相手，活動など）について，場面緘黙児・者を対象とした実験的な研究を推進していくことが必要だろう。

 ・P13　場面緘黙と他の障害との鑑別方法に関して

 ご指摘の通り，FSSMは一部の障害との弁別能が支持されています。FSSMは，場面緘黙と社交不安症および内在化障害との鑑別の有用性が示唆されていました。しかし，場面緘黙と自閉スペクトラム症等の神経発達症との弁別能については未検討でした。この点について，考察3に記載いたしました。

 該当箇所

 Ⅳ．考察　3. 場面緘黙と他の障害との鑑別に用いられた手法　第1段落

 …レビューの結果，場面緘黙と他の障害との弁別能が検証されている評価手法はFSSMのみだった。FSSMは場面緘黙と社交不安症や内在化障害群（うつ病，限局性恐怖症，強迫症，全般不安症，分離不安症，パニック症特定不能の不安症，適応障害）との鑑別については有用性が示唆されていた（Gensthaler *et al*., 2020）。しかし，コミュニケーションの障害を示す点で共通している場面緘黙と自閉スペクトラム症等の神経発達症との鑑別については未検証であり，今後検証されることが望ましい。

 ・場面緘黙と他の障害が併存しているか否かを判断する場合

 場面緘黙と他の障害が併存しているか否かを判断するためには，複数状況下での発話行動の評価による場面緘黙のアセスメントと，併存が疑われる障害のアセスメントの両方を実施し，症状が場面緘黙または他の障害のどちらかだけではうまく説明されないことを確認することが必要であると考えております。場面緘黙と他の障害の併存の判断について，考察3に加筆いたしました。

 該当箇所

 Ⅳ．考察　3. 場面緘黙と他の障害との鑑別に用いられた手法　第1段落

 …場面緘黙と他の障害が併存しているか否かを判断するためには，複数状況下での発話行動の評価による場面緘黙のアセスメントと，併存が疑われる障害のアセスメントの両方を実施し，症状が場面緘黙または他の障害のどちらかだけではうまく説明されないことを確認することが必要であると考えられる。

 ・同質性と異質性に関して

 タイプ分けを行った研究で示されてきたように，場面緘黙には異なるタイプが存在すると考えられます。先行研究で示された異なるタイプの場面緘黙では，緘黙の機能が異なっている可能性が考えられます。中核症状である特定状況下での発話の欠如が共通しているという同質性と，場面緘黙児・者の中でも緘黙の機能は異なっているという異質性，両側面を考慮し，発話評価を進める必要があると考えました。その旨，考察3に記載いたしました。

 該当箇所

 Ⅳ．考察　3. 場面緘黙と他の障害との鑑別に用いられた手法　第2，3段落

 …以上のことから，場面緘黙は異質性のある障害として再度概念化した上で，同質性（homogeneity）と異質性（heterogeneity）の両側面から，評価を進めていくことが必要だと考えられる。具体的には，場面緘黙は，特定状況下で一貫して話していないという症状は共通していてもその機能が異なる可能性が推察される。特定状況下での発話の欠如という共通した症状に対する介入も必要だが，個々の緘黙症状に応じた介入も考えていく必要がある。個に応じた介入を考える上で，機能的アセスメントが有効だと考えられる。例えば，他者からの注目を回避する，負の強化により沈黙する頻度が増加していることもあれば，他者からの援助行動を引き出す，正の強化により沈黙する頻度が増加していることもあるかもしれない。あるいは，特定の場所が先行刺激となり沈黙していることもあるかもしれない。どのような先行刺激によって発話が制御されており，どのような後続刺激によって発話が強化・弱化されているのかを，詳細に検討していくことが，今後必要だろう。

 　場面緘黙の発症に関する遺伝的要因の解明も，場面緘黙と他の障害の鑑別に寄与すると考えられ，今後さらなる研究が求められる。

 ・P13「４．その他の発話評価手法」

 場面緘黙の中核症状が特定状況下での発話の欠如であることから，発話行動の評価が重要であると考え，本研究では発話評価に特に焦点を当てておりました。しかしながら，ご指摘の通り，場面緘黙の介入にあたっては，発話行動以外の行動の抑制についてもアセスメントおよび介入が必要であるため，発話行動以外の行動のアセスメントの必要性について考察4の末尾に加筆いたしました。

 該当箇所

 Ⅳ．考察　4. その他の発話評価手法　第1段落

 　場面緘黙の中核症状は，特定状況における発話の欠如であり，場面緘黙の調査・実験研究において発話行動の評価が重要である。

 Ⅳ．考察　4. その他の発話評価手法　第2段落

 …さらに，場面緘黙児・者では，発話以外の行動の抑制も症状として現れることがあり（河井・河井，1994），介入場面においては，発話以外の行動に関する評価・介入も必要である。FSSMに含まれるように，対象児・者が活動に参加しているかといった項目の評価も，発話行動の評価と併せて実施されることが望まれる。

 その他

 ・題目に関して

 再考しました。診断や診断確定手法，鑑別手法に関する内容を多く含んでいるため，「場面緘黙児・者の診断確定手法を含めた発話評価」に改めることといたしました。

 ・スクリーニングで，著者間で判断に相違があった場合に関して

 判断に相違があったChavira *et al*. (2007) は最終的にレビューに含まれました。その理由に関して結果1に加筆いたしました。

 該当箇所

 Ⅲ．結果　1. レビュー対象論文の選定結果

 …2段階目のスクリーニングにおいて，Chavira, Shipon-Blum, Hitchcock, Cohan, & Stein (2007) をレビューに含めるか否か，著者間で判断に相違があった。Chavira *et al*. (2007) は，主に場面緘黙児の保護者に関する調査だが，場面緘黙児に関するデータも取得していたことから，協議の末，レビューに含めることとした。

 ・測定法が記載しているものの，診断基準が明記されていない論文があることに関して

 ご指摘いただき，記述する必要があると感じましたので，測定法が記載されており診断基準が記載されていない論文があることに関して結果3に加筆いたしました。

 該当箇所

 Ⅲ．結果　3. 場面緘黙の診断確定に用いられた手法　第1段落

 …場面緘黙の診断確定に用いられた測定法について，記載していた研究は44/60編あった。測定法が記載された44編は，医学的診断基準が記載されていた44編とは一致しておらず，測定法が記載されているものの医学的診断基準が明確にされていない研究が11編存在した。診断確定の手法として，複数の方法の併用や医療記録によって総合的に判断した研究が最も多かった（24編）。

 ・Table1でのICD-9の記述に関して

 著者らの調べる限りでは，ICD-9の診断基準については情報が得られませんでした。その旨をTable1の注に記載しました。

 ・Table2に関して

 ご指摘ありがとうございます。Table2に誤りがあり，下8行が欠けておりましたので修正いたしました。

 ・P7L39　FSSMの引用文献に関して

 オンラインで出版されたのが2018年であり，雑誌に収録されたのが2020年でした。

 ・P7L52　群間比較に関して

 群間比較研究の数に関して，記述を改めました。また，場面緘黙以外の障害群との群分けの手法に関して，追記いたしました。

 該当箇所

 Ⅲ．結果　4. 場面緘黙と他の障害との鑑別に用いられた手法　第1段落

 　群間比較を行った研究は47編あり，そのうち，場面緘黙群と定型発達群を比較した研究は20編だった。それ以外の27編は，場面緘黙以外の障害群を対照群として含んでおり，その内訳は次の通りである。まず，社交不安症群を対照群に含む研究は合計で10/27編あり，この10編の中で，社交不安症群のみを対照群とした研究が3編，社交不安症群と定型発達群を対照群とした研究が4編，社交不安症群と内在化障害群（うつ病，限局性恐怖症，強迫症，全般不安症，分離不安症，パニック症特定不能の不安症，適応障害）と定型発達群を対照群とした研究が3編だった。次に，場面緘黙以外の複数の不安症を対照群として設定した研究は合計で10/27編あり，この10編の中で，不安症群のみを対照群とした研究が2編，不安症群と定型発達群を対照群とした研究が7編，不安症群と不安症とADHDの併存群を対照群とした研究が1編だった。その他には，場面緘黙と自閉スペクトラム症の併存群（1/27編），全般不安症群（1/27編），不安症や情緒障害を有する群（小児期の全般性不安障害（ICD-10 F93.80），小児期の情緒障害不特定のもの（ICD-10 F93.9），小児期の社会不安障害（ICD-10 F93.2））（1/27編），情緒障害群（不登校，恐怖反応，適応反応，不安，うつ，夜尿症，ヒステリー）（1/27編），場面緘黙以外の精神障害を有する群（小児期に特異的に発症する情緒障害（ICD-10 F93），特異的会話構音障害（ICD-10 F80.0），表出性言語障害（ICD-10 F80.1））（1/27編）を対照群とした研究が存在した。そして，対照群の障害・疾患が不明な研究が2/27編あった。

 Ⅲ．結果　4. 場面緘黙と他の障害との鑑別に用いられた手法　第2段落

 　社交不安症群を対照群に含む研究（10編）では，場面緘黙群と社交不安症群の群分けのために半構造化面接（8/10編）もしくは質問紙（2/10編）が用いられていた。半構造化面接を実施し場面緘黙群と社交不安症群を群分けした8編の研究では， Kinder-DIPS，ADIS-Ⅳ-C/P， DICA-Ⅳが実施されていた。社交不安症群と内在化障害群（うつ病，限局性恐怖症，強迫症，全般不安症，分離不安症，パニック症特定不能の不安症，適応障害）と定型発達群を対照群とした研究（3編）では，内在化障害群および定型発達群の参加者に場面緘黙または社交不安症の症状が示された場合にはKinder-DIPSにより診断確定が行われたと記載されていた。

 Ⅲ．結果　4. 場面緘黙と他の障害との鑑別に用いられた手法　第3段落

 　場面緘黙以外の不安症群を対照群に含む研究（10編）には，半構造化面接のみによって場面緘黙群と他の不安症群の群分けをした研究が5/10編，半構造化面接と質問紙の併用によって群分けした研究が4/10編，群分けの方法について記載のない研究が1/10編あった。半構造化面接のみを行った研究ではADIS-Ⅳが実施されていた（1編はParent Versionのみ，4編はChild VersionとParent Version）。不安症群と不安症とADHDの併存群を対照群とした研究（1編）では，ADIS-Ⅳ-C/Pによって場面緘黙と判断された対象者が場面緘黙群に分類された。群分けのために一般的な精神保健評価面接によって，現在の問題，発達歴，家族歴，治療歴も確認されたと記載されていた。

 Ⅲ．結果　4. 場面緘黙と他の障害との鑑別に用いられた手法　第5，6，7段落

 　全般不安症群を対照群とした研究（1編）では，Kiddie Schedule for Affective Disorders and Schizophrenia Present and Lifetime Version （K-SADS-PL）によって群分けが行われていた。両障害が併存していた場合の手続きについては記載がなかった。

 　不安症や情緒障害を有する群（小児期の全般性不安障害（ICD-10 F93.80），小児期の情緒障害不特定のもの（ICD-10 F93.9），小児期の社会不安障害（ICD-10 F93.2））を対照群とした研究（1編），情緒障害群（不登校，恐怖反応，適応反応，不安，うつ，夜尿症，ヒステリー）を対象とした研究（1編）では，医療記録を基に群分けが行われていた。場面緘黙と他の障害が併存していた場合の手続きについては記載がなかった。

 　場面緘黙以外の精神障害を有する群（小児期に特異的に発症する情緒障害（ICD-10 F93），特異的会話構音障害（ICD-10 F80.0），表出性言語障害（ICD-10 F80.1））を対象群とした研究（1編）では，児童・青年精神科で受けた診断を基に群分けが行われていた。場面緘黙と他の障害が併存していた場合の手続きについては記載がなかった。

 ・Table 2のADIS-Ⅳに関して

 ADIS-Ⅳは場面緘黙診断確定のために作成された質問が含まれており，太字にすべきでした。太字になっていない箇所があったため修正いたしました。

 ・診断目的で用いられた手法では妥当性検証が行われた手法はFSSMのみだったことに関して

 診断確定以外の目的で使用された発話評価手法は，場面緘黙の症状評価を目的として開発された手法でした。そのため，妥当性検証の際に，場面緘黙症状が妥当に評価できるかという点で検証が行われたのだと考えられます。一方で，診断確定のために使用された評価手法では，FSSM以外の手法は場面緘黙だけでなく，様々な障害の診断を確認することを目的として開発された手法でした。ADIS-Ⅳ，Kinder-DIPS，DICA-Ⅳ，BCFPIでは，評価者間一致率の確認や再検査信頼性の確認など，妥当性検証が実施されていましたが，対象者に場面緘黙児が含まれていなかったために，場面緘黙に関する妥当性検証はできていませんでした。場面緘黙の診断確定を主目的として開発された手法でないために，場面緘黙に関する妥当性検証が欠けてしまったのではないかと考えました。この点について，考察2に加筆いたしました。

 該当箇所

 Ⅳ．考察　2. 場面緘黙の診断確定に用いられた手法　第1段落

 …いくつかの研究において診断確定に使用されたADIS-Ⅳ，Kinder-DIPS，DICA-Ⅳ，BCFPIは，一部の障害に関しては妥当性が検証されていたが，その検証において対象者に場面緘黙児が含まれておらず，場面緘黙に関しては妥当性検証がされていなかった。ADIS-Ⅳ，Kinder-DIPS，DICA-Ⅳ，BCFPIは，様々な障害の診断を目的として開発された手法であり，場面緘黙の診断を主目的としていたわけではないため，場面緘黙に関する妥当性検証が欠けたままになっているのではないかと考えられる。…FSSMは場面緘黙の評価を目的として開発された手法であり，原版のドイツ語版において，定型発達群，社交不安症群，内在化障害群（うつ病，限局性恐怖症，強迫症，全般不安症，分離不安症，パニック症特定不能の不安症，適応障害）との弁別能が支持されている（Gensthaler *et al*., 2020）。

 ・P13L8　「発話行動のを実験的検討」→「を」削除

 ​​​​​​​ご指摘の通り修正いたしました。

# Peer review: Reviewer response for version 1

**DOI:** 10.5256/f1000research.125033.r145718

**Published:** 2022-08-18

**Authors:** Yoshihiro Kanai

**Affiliations:** 1 Faculty of Liberal Arts, Tohoku Gakuin University, Miyagi, Japan

本研究は，場面緘黙児・者の発話評価に関するアセスメントについてPRISMAの手順に従って系統的レビューを行いました。場面緘黙の確定診断，および社交不安症や自閉スペクトラム症など，場面緘黙と併存しやすい疾患との鑑別のために，異なる社会的状況での発話評価が重要と考え，複数の状況における発話の評価がどの程度行われているか整理することが目的でした。その結果，診断のために使用されている面接や質問紙のほとんどは妥当性が検証されていないこと，発話評価に関する研究のほとんど（17編中16編）においては重症度測定のために質問紙が使用されており，行動観察を行った研究は１編のみであったことが明らかにされました。本研究によって，場面緘黙児・者の発話評価に関する現状が明らかになり，今後，必要な研究が明確になりました。また，場面緘黙診断について妥当性が検証された評価方法はFSSM（Frankfurt Scale of Selective Mutism）のみであるという情報や，日本語で使用可能な場面緘黙診断確定方法に関する情報もあり，読者にとっても有益です。

 以下の点について検討されることで，より明確で有益な論文になると思われます。

 ●Major comments

 コメント１　p.12 最終段落

 場面緘黙の診断確定に用いられた手法のうち，行動観察に基づく評価手法が60編中６編であったこと，それぞれの研究が独自の観察を行っていたことが考察で述べられています。これらの行動観察に関する結果については，「結果」のセクションにも記載いただき，今後の研究のためにも，独自に行われていた観察がどのような観点でどのように評価していたのか（考察では，共通しているところと異なるところ；必要な観点があるかどうかなど）を加筆いただければと思います。

 コメント２　p.13　第２段落

 場面緘黙の異質性に関する考察は大変興味深いと思います。これまでの発話評価手法ではいくつかの場面における発話頻度を主として測定していますが，ここにあげられているようにいくつかのタイプがあることを考えると，緘黙することがどのような結果をもたらしているのか，という機能に関するアセスメントも必要であるように思いました。本研究のテーマである発話評価という点と絡めて場面緘黙の異質性について考察していただく観点として機能的アセスメントなどがあげられると思いましたが，いかがでしょうか。著者らのお考えをご教示いただき，必要に応じて加筆いただければと思います。

 コメント３ p.13

 本研究の主要な目的である「複数の状況における発話評価」という点について，p.13「４．その他の発話評価手法」において，実施されていたのかどうかを明記いただければと思います。その前の「３．鑑別に用いられた手法」のところでは「複数状況下での発話行動の評価をアセスメントに含めること」が必要と明記されています。「４．その他の発話評価手法」に関しては，既存の尺度で複数状況における発話評価はできているけれども，行動観察が十分ではない，という結果であったと理解してよろしいでしょうか。

 コメント４

 Table 2など，尺度の略称については，noteなどで何の略かわかるように加筆いただけるとよいと思います。ただし，文量が多くなるため，編集の方針として本文中の記載にとどめるということであればご放念ください。

 ●Minor comments

 要旨　下から２行目

 診断確定ため→診断確定のため

 p.5　「情報の抽出と統合」２行目

 目的としため→目的としたため

 p.14 l.8

 発話行動のを実験的検討は不足している→発話行動の実験的検討は不足している

（該当する場合は要回答）統計分析および解釈は適切ですか。

Yes

システマティックレビューの根拠と目的は明確に示されていますか。

Yes

方法と分析について第三者による再現が可能となるよう十分な詳細が提示されていますか。

Yes

結論はレビューの結果により妥当な裏付けを得ていますか。

Yes

Reviewer Expertise:

臨床心理学，不安症（特に社交不安症），認知行動療法

I confirm that I have read this submission and believe that I have an appropriate level of expertise to confirm that it is of an acceptable scientific standard, however I have significant reservations, as outlined above.

# Response

**Published:** 2022-11-04

**Authors:** 友里亜 藤間

**Affiliations:** 筑波大学大学院, Japan

金井先生

 査読コメントを下さり誠にありがとうございます。

 以下のように加筆・修正を行いました。

 コメント１　p.12 最終段落

 行動観察を実施していた6編の研究のうち5編で家庭場面の録画・録音によって，家庭で話していることが確認されていました。そのうち１編は，家庭場面の録画・録音に加えて，実験室場面の発話行動も確認されました。観察が行われた６編のうち1編は，どのような観察を実施したのか，詳細な記述はありませんでした。診断確定のために観察を実施した６編の研究について結果3に加筆いたしました。また，家庭以外の場面の観察が特に不足しており，今後，複数状況の観察が期待されるという旨を考察2に加筆いたしました。

 該当箇所

 Ⅲ．結果　3. 場面緘黙の診断確定に用いられた手法　第1段落

 …場面緘黙診断確定手法として確立された観察手続きはなかったが，発話行動の観察は診断確定のために重要であると考え，観察実施の有無と観察の内容についても確認した。

 Ⅲ．結果　3. 場面緘黙の診断確定に用いられた手法　第6段落

 　各研究独自の観察が診断確定に含まれていた研究が6/60編存在した（Arie *et al*., 2007; Bar-Haim *et al*., 2004; Henkin, Feinholz, Arie, & Bar-Haim, 2010; Kristensen, 2000; Kristensen & Oerbeck, 2006; Muchnik *et al*., 2013）。6編すべての研究において，観察と観察以外の手法を組み合わせて診断確定が行われていた。Bar-Haim *et al*.（2004），Henkin *et al*.（2010）， Muchnik *et al*.（2013）で実施された観察では，家庭場面の録画・録音によって，話す能力のあることが確認された。Kristensen（2000）では，家庭場面の録音によって，言語スキルが評価された。Arie *et al*.（2007）では，家庭場面の録画・録音による話す能力の確認に加え，実験室での保護者および見知らぬ実験者との相互作用中の発話行動も観察された。Kristensen and Oerbeck（2006）は，診断確定の手続きに直接観察が含まれていたと記載されていたが，どのような場面の観察を実施したのかについて詳細な記載はなかった。

 Ⅳ．考察　2. 場面緘黙の診断確定に用いられた手法　第2段落

 …面接や質問紙の標準化に加えて，行動観察に基づく評価手法の開発も今後の課題である。複数の評価指標や情報に基づき，総合的に場面緘黙の診断を下すことを提唱しているガイドライン（Dow, Sonies, Scheib, Moss, & Leonard, 1995）においても，複数の社会的状況下での発話行動の実測については言及されていない。しかし，質問紙や半構造化面接で扱われている内省的な報告は，実際の行動と乖離する可能性が，これまでの研究で問題点として指摘されている（Baumeister,  Vohs, & Funder, 2007）。質問紙や面接だけでなく，行動観察による発話評価を併せて行うことが妥当性の高い診断確定には必要だろう。今後の研究では，場面緘黙児・者の発話の観察を含めた評価手法の確立が望まれる。レビュー対象の論文でも観察を行った研究は少ないながらも存在しており（6/60編），5/6編において家庭場面で問題なく話していることが確認されていた。1/6編の研究では，家庭場面の観察に加えて，家庭以外の場面の観察も実施された。ただし，それぞれの研究が独自の観察を行っており，場面緘黙診断確定のために確立された観察法はなかった。また，これまでの調査・実験研究では，一貫して話していない状況の観察が特に不足していた。場面緘黙児の多くは学校で話すことができないため（Rodrigues Pereira *et al*., 2021），学校場面の録画を提出するよう求めることが有用だと考えられるが，普段の授業時間や休み時間など，児童・生徒が多数いる状況を録画することについて同意を得るのは手続き上の困難があると推察される。場面緘黙当事者と，特定の話すことができない相手（例えば，担任教員）の同意を得て，同意が得られた人々だけの状況を設定してもらい，その状況を録画し提出するよう依頼するのが現実的かもしれない。このように設定された状況は，一貫して話していない普段の状況とは異なってしまうが，話していない状態は確認できる。…Arie *et al*.（2007）で実施されていたように複数状況での観察を行うことが望ましいと考えられる。複数状況での発話行動を定量的に評価し・比較し，状況によって発話行動が顕著に異なることを確認できるような観察手続きやチェック項目が確立されると，場面緘黙児・者を対象とした研究の参加者を選定する際に有用である。これまでの調査・実験研究では，観察を含む診断確定を行った研究は少なかった（6編）が，手法の確立によって，観察を実施する研究の増加が期待される。

 コメント２　p.13 第2段落

 ご指摘の通り，機能的アセスメントは場面緘黙を理解するうえで重要な視点だと感じました。発話の欠如という症状は共通していても，様々な機能があると考えられます。また，後続刺激だけでなく，先行刺激も緘黙症状の制御に関わっていると考えました。機能的アセスメントに関して，考察3に加筆いたしました。

 該当箇所

 Ⅳ．考察　3. 場面緘黙と他の障害との鑑別に用いられた手法　第2段落

 …以上のことから，場面緘黙は異質性のある障害として再度概念化した上で，同質性（homogeneity）と異質性（heterogeneity）の両側面から，評価を進めていくことが必要だと考えられる。具体的には，場面緘黙は，特定状況下で一貫して話していないという症状は共通していてもその機能が異なる可能性が推察される。特定状況下での発話の欠如という共通した症状に対する介入も必要だが，個々の緘黙症状に応じた介入も考えていく必要がある。個に応じた介入を考える上で，機能的アセスメントが有効だと考えられる。例えば，他者からの注目を回避する，負の強化により沈黙する頻度が増加していることもあれば，他者からの援助行動を引き出す，正の強化により沈黙する頻度が増加していることもあるかもしれない。あるいは，特定の場所が先行刺激となり沈黙していることもあるかもしれない。どのような先行刺激によって発話が制御されており，どのような後続刺激によって発話が強化・弱化されているのかを、詳細に検討していくことが，今後必要だろう。

 コメント３　p.13

 その他の発話評価に関して，複数の状況における発話評価が実施された研究と学校場面だけの評価が実施された研究がありました。ご指摘の通り，質問紙や面接によって，複数状況における発話評価を行っている研究はありますが，行動観察が少ないという結果でした。結果5，考察4に加筆いたしました。

 該当箇所

 Ⅲ．結果　5. その他の発話評価手法　第2段落

 　保護者回答の質問紙（14編）には，Selective Mutism Questionnaire（SMQ）（10/14編），FSSMの重症度尺度（1/14編）があった。いずれも，複数状況下での発話行動を評価する質問紙だった。

 Ⅲ．結果　5. その他の発話評価手法　第3段落

 　…DortMus-Kitaは，学校場面に限定された発話行動を尋ねる質問紙だが，DortMus-Kitaを使用したStarke（2018）は，保護者を対象としたオリジナルの質問紙を併用し，家庭や近所，公共の場での発話行動についても評価していた。…School Speech Questionnaire を使用したレビュー対象の研究（Bergman *et al*., 2002）では，学校以外の状況での発話行動については評価されていなかった。また，Bergman *et al.* (2002) では， School Speech Questionnaireのうち，項目テスト相関の低かった2項目を除外した9項目が使用されていた。

 Ⅲ．結果　5. その他の発話評価手法　第4段落

 …TTI-SMは，学校場面に限定された発話行動について尋ねる面接だが，TTI-SM を使用した研究（Martinez *et al*., 2015）は，家庭，学校，その他の社会的状況での発話行動を尋ねる保護者回答の質問紙であるSMQを併せて実施していた。

 Ⅲ．結果　5. その他の発話評価手法　第5段落

 　観察により発話評価した研究（Edison *et al*., 2011）では，実験室での場面緘黙児とその保護者の会話場面を録画し，複数状況下での子どもの発話と保護者の発話について観察者が評価した。

 Ⅳ．考察　4. その他の発話評価手法　第2段落

 …質問紙や面接を用いて複数状況下での発話評価を行った研究はあったものの，複数状況下での観察を行い，実際の発話を定量的に測定した研究は，レビュー対象の論文のうち，1編のみだった（Edison *et al*., 2011）。

 コメント４

 Table2，3に注を加えました。

 Minor comments

 ご指摘いただいた箇所を修正いたしました。

# Peer review: Reviewer response for version 1

**DOI:** 10.5256/f1000research.125033.r145713

**Published:** 2022-08-16

**Authors:** Shin-ichi Ishikawa

**Affiliations:** 1 Faculty of Psychology, Doshisha University, Kyoto, Japan

本論文は，場面緘黙児・者に対する評価方法を体系的に展望することを目的としています。著者らが本論文内で指摘しているように，場面緘黙について確立された評価方法が少ないという現状を踏まえると，本展望論文のもたらす情報は臨床的意義が高いと考えます。いくつかのコメントをさせていただきましたので，改稿の参考にしていただければ幸いです。

 メジャーなコメント

 １．診断基準の変遷について

 場面緘黙がDSMやICDのバージョンによって異なる点は理解できました。しかし，それは全ての障害に当てはまるものかと思いますし，ある意味避けようのないことかと思います。もし特定の議論をする際に，許容できる範囲を超えて診断基準間の不統一が問題なのであれば，むしろ１つの基準で診断された研究のみを中心にレビューすべきかと思います。あるいは，たとえばASDや不安症などの変遷と比較しながら，場面緘黙特有の問題があれば論じていただければ意義深いかと思います。

 ２．鑑別診断について

 おそらく場面緘黙は，他の障害と併存することもあるのだと思います。当該の議論には，もしクライエントが２つの障害を同時に持っているとして，①ある特定の障害がマスクしてしまっているケース，②適切に２つ以上の障害の併存を見極めているケース，があるかと思います。加えて，併存症はなく場面緘黙のみ純粋に有しているにもかかわらず，③誤って２つの障害を重複してつけてしまっているケースもあるかと思います（たとえば，場面緘黙と社交不安症）。このあたり，診断にかかる議論を整理して論じていただければと思います。その上で，７ページの結果の箇所は，診断基準自体の不統一性に焦点を当てているのか，それとも場面緘黙自体の特徴から，①や③の鑑別診断の難しさを述べているのか，明確にしていただければと思います。たとえば，本論文のTable 1に従いますと，ICD-10に基づいていればASDとの併存はないけれども，DSM-5であれば，その可能性を含むということは至極当然のように思います。上記の診断基準の変遷と合わせて議論すべき点を整理していただければ幸いです。また，下記３のコメントにも関連するのですが，どのような対象者に対して鑑別診断を試みているのかが，不統一のために議論が整理されづらくなっていると思います。対象者全員が場面緘黙であることが確定しているのであれば，その中から併存診断や鑑別診断をするのは分かりますが， 結果２をみると16研究は医学的診断基準がないとなっています。医学的診断のある研究と質問紙で判定した研究を同質としてシステマティックレビューに含める点は議論が残るかもしれません。そのため，対象となった研究を明確に定義していただくと，このあたりの解釈が分かりやすくなるかと思います。

 ３．対象となる研究について

 今回対象にしている研究は，場面緘黙を対象としてケースコントロールデザインを用いた観察研究という認識で良いのでしょうか（もしそうであれば，横断，縦断，回顧的などの情報もあると有益かもしれません）。いずれにせよ，選定基準に除外基準はあるのですが，結局どのような論文を含んでいるのかという包含基準が分かりませんでした。たとえば，全ての研究はどこかの段階で場面緘黙の診断プロセスを経ていて，そのことについて「3. 場面緘黙の診断確定に用いられた手法」で整理されており，その他の測定が「5. その他の発話評価手法」ということであれば，そのあたりを論文の選定基準に記載いただければと思います。ただ，そう考えると，コメント２にも関連して，７ページの「診断を確定する測定法として面接(22 編)，質問紙(7 編)が用いられていた。」という結果が少し不思議に思えます。質問紙を用いて“診断”した結果が，本研究の合致基準として適切かどうか，という疑問が残るからです（つまり，アナログ研究の可能性は否定できるのか，という疑問です）。単純に，「3. 場面緘黙の診断確定に用いられた手法」と「5. その他の発話評価手法」は，（本論文で新たに基準を設けているのではなく）各研究で独自に設定している独立変数と従属変数ということなのでしょうか。加えて，介入研究を加えなかった理由を教えてください。頑健なトライアルでは診断基準などを用いて，場面緘黙の対象者を選抜した上で，効果測定のための指標を複数用いていると思いましたので，本研究のレビューの目的に合致するのではないかと考えました。

 ４．妥当性について

 さまざまな評価法について，妥当性がないことを問題として取り上げていますが，その際の妥当性が何を表しているのか具体的に記述いただければと思います。たとえば，質問紙であれば併存的妥当性などを指しているのではないかと推測しますが，診断面接などはどのような妥当性を想定しているのでしょうか。おそらく各評価手法によって，検証すべき妥当性の領域も異なってくるかと思います。また，必要に応じて，信頼性についても加筆いただければと思います。

 ５．「5. その他の発話評価手法」について

 上記コメント３とも関連しますが，このカテゴリが各論文によるものなのか，本研究での定義に基づいて抽出されたものであるのか，必要に応じて加筆修正をいただければと思います。FSSMのように「3. 場面緘黙の診断確定に用いられた手法」と一部重複があるので，それぞれについて，「4. 情報の抽出と統合」で示していただいている記述に基づいて，本研究での明瞭な定義を示していただければと思います。

 マイナーなコメント

 ４ページ：２段落で述べている併存率のばらつきがあまりに大きいので，分散等も併記いただいた方が良いかもしれません。

 ８ページ：不安「障害」にここはなっています。

 11ページ：T単位について追加の説明をしていただけますか。

 以上です。

（該当する場合は要回答）統計分析および解釈は適切ですか。

Not applicable

システマティックレビューの根拠と目的は明確に示されていますか。

Yes

方法と分析について第三者による再現が可能となるよう十分な詳細が提示されていますか。

Partly

結論はレビューの結果により妥当な裏付けを得ていますか。

Yes

Reviewer Expertise:

臨床児童心理学

I confirm that I have read this submission and believe that I have an appropriate level of expertise to confirm that it is of an acceptable scientific standard, however I have significant reservations, as outlined above.

# Response

**Published:** 2022-11-04

**Authors:** 友里亜 藤間

**Affiliations:** 筑波大学大学院, Japan

石川先生

 査読コメントを下さり誠にありがとうございます。

 以下のように加筆・修正を行いました。

 １．診断基準の変遷について

 診断基準の変遷に触れたのは，発話評価に着目する意義を示すという意図がありました。場面緘黙は診断基準によって一部基準が異なっており，対象としている集団が異なる可能性があります。しかし，状況によって発話行動が異なっており，特定の状況で発話が欠如していることを確認する必要があるという点は共通していました。そのため，どの診断基準に基づいても，場面緘黙症状の評価として，複数状況での発話評価が必要になります。したがって，発話評価手法についてのレビューに関しては，異なる診断基準を使用した研究を対象として問題ないと考えました。

 　本研究の主目的は，発話評価手法についてレビューすることだったため，診断基準の変遷に関しては，場面緘黙症状の評価に関わる補足的な内容として位置づけました。意図が明確になるように，序論の最終段落および考察１の記述を修正いたしました。

 該当箇所

 Ⅰ．序論　第4段落

 …しかし，他の診断との鑑別がどのように行われたかについての検討はされていなかった。本研究の目的は，場面緘黙児・者を対象とした調査・実験研究において，場面緘黙診断の確定方法や，場面緘黙と他の障害との鑑別手法も含め，発話がどのように評価されてきたかを整理することだった。また，レビュー結果に基づき，異なる社会的状況での発話について，信頼性の高い客観的な評価を行うための課題について考察することも目的とした。以上の目的に関連して，場面緘黙の診断基準についても，その変遷を整理した。

 Ⅳ．考察　1. 用いられた医学的診断基準

 　用いられた診断基準には，ICD-9，ICD-10（World Health Organization, 1993;　中根・岡崎・藤原・中根・針間訳，2008），DSM-Ⅲ（American Psychiatric Association, 1980），DSM-Ⅲ-R（American Psychiatric Association, 1987），DSM-Ⅳ（American Psychiatric Association, 1994），DSM-Ⅳ-TR（American Psychiatric Association, 2000），DSM-5（American Psychiatric Association, 2013）があった。ICD-9では，精神疾患に起因しない広範かつ持続的な発話の拒否という記述が認められたものの（National Center for Health Statistics, 1980, p.1091），診断基準について詳細な情報は，著者らの調べる限りでは得られなかった。DSM-Ⅳ，DSM-Ⅳ-TR，DSM-5の診断基準は同一だが，これらの診断基準以外の診断基準では，社会的状況の種類や症状の持続期間など，それぞれ異なる点が存在している。例えば，DSM-Ⅲ，DSM-Ⅲ-Rでは，学校で話せないこと，多くの状況で話せないことが条件になっているが，他の診断基準では，話せない状況が学校である必要はなく，話せない状況が複数である必要もない。DSM-Ⅲ，DSM-Ⅲ-Rでは，症状の持続期間に関する明確な基準はないが，DSM-Ⅳ，DSM-Ⅳ-TR，DSM-5では症状が1か月以上，ICD-10では4週間以上持続することが診断基準に含まれている。言語表現及び言語理解について，ICD-10では，標準化検査の得点が2標準偏差以内であることが基準として記載されているが，DSMはすべての版において明確な言語能力の基準はない。診断基準が異なる場合，個々の研究結果の解釈や比較をする際には，個々の研究で用いた診断基準を考慮する必要がある。一方で，診断基準ごとに社会的状況の種類や症状の持続期間などに違いはあるものの，話すことができる社会的状況と話すことができない社会的状況の両方があるという点はすべての診断基準に共通していた。したがって，どの診断基準を使用する場合であっても，場面緘黙診断確定および場面緘黙症状の測定のためには複数の社会的状況下での発話行動を評価する必要がある。

 ２．鑑別診断について

 場面緘黙と他の障害との鑑別に関して，おっしゃる通りの実態があり，場面緘黙のみを有している場合に場面緘黙のみの診断を下すこと，他の障害のみを有している場合に場面緘黙の診断を下さないこと，場面緘黙と他の障害が併存している場合に併存診断を下すことが適切にできることが求められると考えます。鑑別のために複数状況下での発話行動の評価が必要であると考えております。また，場面緘黙と他の障害が併存しているか否かを判断するためには，複数状況下での発話行動の評価による場面緘黙のアセスメントと，併存が疑われる障害のアセスメントの両方を実施し，症状が場面緘黙または他の障害だけでうまく説明されないことを確認することが必要であると考えられます。この旨を考察3に加筆しました。

 　場面緘黙と他の障害との鑑別診断に関して，「診断基準の不統一性」ではなく「場面緘黙の特徴による鑑別診断の難しさ」に焦点を当てました。そこで，結果4では，場面緘黙と他の障害との鑑別に用いられた手法は研究ごとに異なっており，その手法は確立されていないという旨を補足しました。

 　レビュー対象となった研究がどのような研究だったのか，方法2および結果1に加筆いたしました。レビュー対象となった研究は，場面緘黙児・者を対象とした調査・実験研究でした。他の障害群を対照群に含んでいた研究は27編であり，この27編について，場面緘黙と他の障害との鑑別手法をレビューしました。

 該当箇所

 Ⅱ．方法　2. 論文の選定基準

 　英語で記述された場面緘黙児・者を対象とした実証データに基づく調査・実験研究をレビューの対象とした。介入研究は多く実施されており，行動療法や認知行動療法，薬物療法が有効であるなど，介入に関する知見が蓄積されている（ *e.g*., Cohan *et al*., 2006; Manassis *et al*., 2016）一方で，場面緘黙児・者がどのような特徴を有している集団なのかといった情報が不足しており，調査・実験研究が更に必要である。今後，調査・実験研究を進めるにあたり，これまでの調査・実験研究における評価手法に関するレビューが有用だと考え，本研究では，調査・実験研究を対象とした。英語以外の言語で記述された文献，展望論文，質的研究，疫学研究，介入研究は除外した。

 Ⅲ．結果　1. レビュー対象論文の選定結果　第2段落

 　対象となった論文は，場面緘黙児・者のみを対象とした調査・実験研究が13編，対照群との比較を行った調査・実験研究は47編だった。群間比較研究には，定型発達群のみを対照群とした研究が20編，定型発達以外の群を対照群に含む研究が27編あった。

 Ⅲ．結果　4. 場面緘黙と他の障害との鑑別に用いられた手法　第１段落

 …鑑別方法が記載された研究間では，その方法が異なっており，場面緘黙と他の障害との鑑別方法が確立されていなかった。

 Ⅳ．考察　3. 場面緘黙と他の障害との鑑別に用いられた手法　第1段落

 …したがって，複数状況下での発話行動の評価をアセスメントに含めることで，他の障害との鑑別が可能になるかもしれない。場面緘黙と他の障害との鑑別に関して，場面緘黙のみを有している場合に場面緘黙のみの診断を下すこと，他の障害のみを有している場合に場面緘黙の診断を下さないことに加えて，場面緘黙と他の障害が併存している場合に併存診断を下すことが適切にできることが求められる。場面緘黙と他の障害が併存しているか否かを判断するためには，複数状況下での発話行動の評価による場面緘黙のアセスメントと，併存が疑われる障害のアセスメントの両方を実施し，症状が場面緘黙または他の障害だけでうまく説明されないことを確認することが必要であると考えられる。

 ３．対象となる研究について

 対象となった研究は，場面緘黙児・者のみを対象とした調査・実験研究が13編，対照群との比較を行った調査・実験研究は47編でした。群間比較研究には，定型発達群のみを対照群とした研究が20編，定型発達以外の群を対照群に含む研究が27編ありました。対象となった研究について結果1に加筆いたしました。

 　介入研究は多く実施されており，行動療法や認知行動療法，薬物療法が有効であるなど，介入に関する知見が蓄積されている一方で，場面緘黙児・者がどのような特徴を有している集団なのかといった情報が不足しており，調査・実験研究が更に必要であると考えています。今後，調査・実験研究を進めるにあたり，これまでの調査・実験研究における評価手法に関するレビューが有用だと考え，本研究では，調査・実験研究を対象としました。この点について方法2に加筆いたしました。

 該当箇所

 Ⅱ．方法　2. 論文の選定基準

 …介入研究は多く実施されており，行動療法や認知行動療法，薬物療法が有効であるなど，介入に関する知見が蓄積されている（e.g., Cohan *et al*., 2006; Manassis *et al*., 2016）一方で，場面緘黙児・者がどのような特徴を有している集団なのかといった情報が不足しており，調査・実験研究が更に必要である。今後，調査・実験研究を進めるにあたり，これまでの調査・実験研究における評価手法に関するレビューが有用だと考え，本研究では，調査・実験研究を対象とした。

 Ⅲ．結果　1. レビュー対象論文の選定結果　第２段落

 　対象となった論文は，場面緘黙児・者のみを対象とした調査・実験研究が13編，対照群との比較を行った調査・実験研究は47編だった。群間比較研究には，定型発達群のみを対照群とした研究が20編，定型発達以外の群を対照群に含む研究が27編あった。

 ４．妥当性について

 妥当性検証が行われている尺度については，どのような検証が行われていたのかを加筆いたしました。信頼性の指標の1つである再検査信頼性は妥当性を支持する1つの証拠として扱いました。内的一貫性は，妥当性の観点からの解釈が困難だと考え，本研究では取り上げませんでした。評価手法の種類にかかわらず，目的とする構成概念を測定できているかの検証は必要だと考えます。診断確定手法については，特に，場面緘黙児・者と非場面緘黙児・者の弁別能が検証されているかが重要だと考えます。

 該当箇所

 Ⅲ．結果　3. 場面緘黙の診断確定に用いられた手法　第１段落

 …場面緘黙診断を目的として開発された測定法には，面接と質問紙があり，面接が22編，質問紙が7編で用いられていた。場面緘黙診断確定を目的として開発された測定法について，妥当性検証の有無を確認した。再検査信頼性は信頼性の指標として，妥当性と区別して扱われる場合もあるが，村山（2012）を参考に，本研究では，妥当性を支持する証拠の1つとして扱った。また，信頼性の指標として，内的一貫性も挙げられる。しかし，内的一貫性が高すぎると，構成概念を網羅する幅広い項目が尺度に含まれていないことを示唆し，内的一貫性と妥当性の両立は困難であると指摘されている（村山，2012）。したがって，内的一貫性は妥当性の観点からの解釈が難しいと考え，本研究では妥当性の証拠として取り上げなかった。

 Ⅲ．結果　3. 場面緘黙の診断確定に用いられた手法　第2段落

 …ADIS-Ⅳ-P/CはLyneham, Abbott, and Rapee (2007)，Silverman, Saavedra, and Pina (2001)，Wood, Piacentini, Bergman, McCracken, and Barrios (2002）で，一部の障害診断確定に関して妥当性検証は行われていた。具体的には，Lyneham *et al*. (2007) では，分離不安症，全般不安症，社交不安症，限局性恐怖症，強迫性障害について，評価者間一致率が確認された（ *κ* = .80 - 1.0）。Silverman *et al*. (2001) では，分離不安症，社交不安症，限局性恐怖症，全般不安症について，再検査信頼性が確認された（ *κ* = .63 - .80）。また，Wood *et al*.（2002）では，社交不安症，分離不安症，パニック症について，ADIS-Ⅳ-P/C による分類とMultidimensional Anxiety Scale for Children（MASC; March, 1997）の結果に収束性が示唆された。これらの研究では対象者の中に場面緘黙と診断された人々が含まれておらず，場面緘黙診断確定の妥当性は確認されていなかった。

 Ⅲ．結果　3. 場面緘黙の診断確定に用いられた手法　第3段落

 …ただし，レビュー対象となったKinder-DIPSを使用した研究では保護者版のみが実施されていた。…Kinder-DIPS， BCFPI，DICA-Ⅳは一部の障害診断確定に関して妥当性について報告されていた。妥当性評価として，Kinder-DIPSの保護者版では，注意欠如・多動症，夜尿症，うつ病，分離不安症，限局性恐怖症，社交不安症について，評価者間一致率（ *κ* = .74 - .96， *Yules’Y* = .98 - 1.00）が確認されたと報告されている（Adornetto *et al*., 2008）。BCFPIは確認的因子分析の結果，おおむね良好なモデル適合度（GFI = .880 - 904，CFI = .860 - 868，RMSEA = .038 - .056）が得られた（Cunningham *et al*., 2009）。DICA-Ⅳは，De la Osa *et al*.（1997）によって，医師の診断との一致率やCBCLの結果との収束性一致率が良好であることが確認され，また，再検査信頼性（6-12歳用： *κ* = .32 - .65，13-18歳用： *κ* = .59 - .92）の検証も行われたことが報告されている（Reich, 2000）。これらの研究では対象者の中に場面緘黙と診断された人々が含まれておらず，場面緘黙診断確定の妥当性は確認されていなかった。

 Ⅲ．結果　3. 場面緘黙の診断確定に用いられた手法　第5段落

 …FSSM診断尺度はGensthaler *et al*. (2020) で妥当性検証が行われた。妥当性の検証として，ROC解析が行われ，場面緘黙群と定型発達群，社交不安症群，内在化障害群（うつ病，限局性恐怖症，強迫症，全般不安症，分離不安症，パニック症特定不能の不安症，適応障害）との弁別能が確認された（AUC = 0.97- 1.00）。

 Ⅲ．結果　5. その他の発話評価手法　第2段落

 …SMQは学校，家庭，その他の社会的状況の発話場面の項目（ *e.g.*, たいていの同輩と学校で話す，他の人がいても家で家族と話す，医師や歯科医と話す）における発話頻度を，４件法で評価する尺度であり，Bergman, Keller, Piacentini, and Bergman (2008) によって，標準化，妥当性検証が行われている。標準化の手続きとして，Bergman *et al*. (2008) 研究1において主成分分析が行われ，3つの下位尺度からなる合計17項目の尺度となった。Bergman *et al*. (2008) 研究2では，場面緘黙群と場面緘黙以外の不安症群との比較の結果，有意な群間差が示され，妥当性が支持された。…Gensthaler *et al*.（2020）で妥当性検証が行われ，臨床医による場面緘黙重症度評価とFSSM重症度得点に有意な相関が示された（ *r* = .48 - .72）。

 Ⅲ．結果　5. その他の発話評価手法　第3段落

 …妥当性検証が行われており，場面緘黙群と非場面緘黙群の得点を比較し，有意な群間差が示されたと報告されている（Starke & Subellok, 2018）。

 Ⅲ．結果　5. その他の発話評価手法　第4段落

 …Martinez *et al*. (2015）により，妥当性を支持する証拠が得られた。妥当性検証として相関分析が行われ，ADIS-Pによる臨床診断，SMQとの間に有意な相関が示された。また，他の不安症の症状を評価する質問紙である，SASC-RおよびMASCの全体得点との相関が有意でないことが示された。さらに，場面緘黙群と場面緘黙以外の不安症群との比較の結果，有意な群間差が認められた。

 Ⅳ．考察　2. 場面緘黙の診断確定に用いられた手法

 …特に，場面緘黙児・者と非場面緘黙児・者の弁別能に関する妥当性の検証が重要であると考えられる。

 ５．「５．その他の発話評価手法」について

 対象者が場面緘黙に当てはまるか否かを判断するために使用された手法を「場面緘黙の診断確定に用いられた手法」，従属変数として研究中で使用された手法を「その他の発話評価手法」としました。この点について方法4に加筆いたしました。

 　FSSMは診断尺度と重症度尺度の２つの下位尺度で構成されており，診断確定には診断尺度，その他の評価には重症度尺度が使用されていました。Table2，3に下位尺度の別を加筆いたしました。また，SMQもTable2，3に重複しています。SMQは場面緘黙の重要度を評価する尺度であり，場面緘黙の診断確定を目的として作成された尺度ではありません。しかし，他の手法と組み合わせて，診断確定の手続きで使用したと記載されていた研究があり，Table2の診断確定の列に細字で記載しました。この点については結果3に加筆いたしました。

 該当箇所

 Ⅱ．方法　4. 情報の抽出と統合

 …発話評価手法に関しては，対象者が場面緘黙に当てはまるか否かを判断するために使用されたと論文中に記載されている手法を（２）場面緘黙診断確定手法，従属変数として使用されたと論文中に記載されている手法を（４）その他の発話評価手法とした。

 Table2　3，4行目

 FSSM 診断尺度

 Ⅲ．結果　3. 場面緘黙の診断確定に用いられた手法　第１段落

 …SMQは場面緘黙の重症度を評価する尺度だが，診断確定手続きに使用したと記載された研究があり，診断確定の列にも含まれている。診断確定を目的とした測定法ではないため，診断確定の列には細字で示した。

 Table3　2行目

 FSSM 重症度尺度

 マイナーなコメント

 ご指摘いただいた箇所について，加筆・修正いたしました。

 該当箇所

 Ⅰ．序論　第2段落

 …平均69％（95%CI = 0.52, 0.84）だった

 Ⅲ．結果　5. その他の発話評価手法　第5段落

 …発話はT単位に分割された。T単位はHunt （1966）によって定義された，文として成立する最短の単位である。1つの主節，あるいは1つの主節に従属節や埋め込まれた節が加わったものを1単位とする。例えば，“They tried and tried but while they were trying they killed a whale and used the oil for the lamps they almost caught the white whale.”という文は，“They tried and tried.”，“But while they were trying they killed a whale and used the oil for the lamps.”，“They almost caught the white whale”の3つのT単位に分割される（Hunt, 1966）。
